# NaCl stress-induced transcriptomics analysis of *Salix linearistipularis* (syn. *Salix mongolica*)

**DOI:** 10.1186/s40709-016-0038-7

**Published:** 2016-02-29

**Authors:** Guixian Nan, Yan Zhang, Song Li, Imshik Lee, Tetsuo Takano, Shenkui Liu

**Affiliations:** Laboratory of Saline-Alkali Vegetation Ecology Restoration in Oil Field (SAVER), Ministry of Education, Alkali Soil Natural Environmental Science Center (ASNESC), Northeast Forestry University, Hexing Road No. 26, Xiangfang, Harbin, 150040 Heilongjiang China; College of Bioinformatics Science and Technology, Harbin Medical University, Harbin, 150081 China; Institute of Physics, Nankai University, Nankai District, Tianjin, 300071 China; Asian Natural Environment Science Center (ANESC), The University of Tokyo, Midori Cho 1-1-1, Nishitokyo, Tokyo 188-0002 Japan; College of Agriculture, Yanbian University, Yanji, 133002 China

**Keywords:** *Salix linearistipularis*, RNA-Seq, Biological pathway, Salt stress, Differently expressed genes (DEGs), Non differently expressed genes (N-DEGs)

## Abstract

**Background:**

*Salix linearistipularis* (syn. *S. mongolica*) is a woody halophyte, which is distributed naturally in saline-alkali soil of Songnen plain, Heilongjiang, China. It plays an important role in maintaining ecological balance and in improving saline soil. Furthermore, *S. linearistipularis* is also a genetic resource; however, there is no available information of genomic background for salt tolerance mechanism. We conducted the transcriptome analysis of *S. linearistipularis* to understand the mechanisms of salt tolerance by using RNA-seq technology.

**Results:**

The transcription profiles of both the salt stress (SLH-treated) and the control (SLH-control) sample for *S. linearistipularis* were obtained by using RNA-seq in this study. By comparative analysis, only 3034 of 53,362 all-unigenes between two samples were expressed differently at more than 1.5-fold ($\left| {fold - change} \right| \ge 1.5$, FDR ≤ 0.05), including 1397 up-regulated genes and 1637 down-regulated genes. In total, 2199 genes were classified into 50 Gene Ontology (GO) terms and 1103 genes were involved in 116 biological pathways. To find salt stress related genes, all-unigenes of *S. linearistipularis* were classified into three categories according to their degree of the differentially expressed genes (DEGs) at 0–1.5-fold (non differently expressed genes, N-DEGs), at 1.5–4.0-fold and more than 4.0-fold. The pathways of three categorized genes were compared with the DEGs of *Arabidopsis thaliana*, showing that 22, 10 and 1 pathway of *S. linearistipularis* were overlapped with *A. thaliana.* Degree of the overlapping was categorized as 0–1.5-fold, 1.5–4.0-fold and more than 4.0-folds.

**Conclusion:**

Our study revealed that the N-DEGs of 22 pathways in *S. linearistipularis* were overlapped with the DEGs of *A. thaliana*. This result suggests that those overlapped genes that contrasted with the up- or down-regulated genes in *A. thaliana* were possibility evolved into housekeeping genes in *S. linearistipularis* under salt stress.

**Electronic supplementary material:**

The online version of this article (doi:10.1186/s40709-016-0038-7) contains supplementary material, which is available to authorized users.

## Background

Ecological problems of soil salinization have negative effects on local economic and social development. According to the FAO/UNESCO incomplete statistics, there are approximately 4.0 × 10^8^ ha saline soils worldwide, while ~3.6 × 10^7^ ha are located in China [1]. Due to environment and human-induced soil degradation, area of soil salinization increases gradually. Salt stress provokes osmotic stress and ion toxicity, causing excessive ion accumulation. Salt stress-induced oxidative stress results from excess reactive oxygen species (ROS) formation that damages the lipids of the plant cell membrane, proteins and DNA [2]. The long exposure of the plants to the extreme environments (such as drought, cold, high salinity, and other extreme environments) results in the development of corresponding adaptation mechanisms. The corresponding adaptation mechanism appears as the changes of stress-signal perception and transduction, plant morphology, physiology, biochemistry, protein and gene, and the corresponding changes leads inactivating the protection metabolic pathways [3].

When plants expose to soil salinization, the osmotic adjusting matters and the osmotic protecting proteins are induced for ion uptake, ion compartmentalization and antioxidant enzyme synthesis. Osmotic adjustment mechanism in plants is divided into organic and inorganic osmotic adjustments. Inorganic osmotic adjustment is to absorb large amounts of inorganic salts and accumulate the absorbed salt ions into the vacuole, for reducing cell potential to adapt salt stress induced low extracellular water potential. Inorganic ion adjustments in the organ, tissue or cell, reduce ion toxicity by distribution and localization. The small organic solutes including proline, glucose, amino acids, betaine, polyamines, glycerol, sorbitol, inositol, and other small molecules of organic solutes also cause to increase the osmotic pressure of the cell and to reduce water potential. Under salt stress, there are protective enzyme system and antioxidant system for scavenging ROS in plant. Protective enzyme system for defending antioxidant activities in plants mainly involves superoxide dismutase (SOD), catalase (CAT), peroxidase (POD) and glutathione reductase (GR) [4]. Antioxidant system consists primarily ascorbic acid (ASA), reduced glutathione (GSH) and carotenoids. When salt stress triggers the acceleration of the scavenging activity of ROS for maintaining ion homeostasis and protecting chloroplast functions, plants can survive under high salt concentration. Recently, diverse salt tolerance mechanisms that are generated by trans-membrane transportation of small molecules were found in plants. Such molecules are late embryogenesis abundant protein (LEA), osmosis protein (OSM), aquaporin, K^+^ channel protein, ATPase, etc. [5]. Salt stress-generated signals may act as certain common regulatory factors. These regulatory factors control the salt-induced gene expressions. Up-to-date, significant progresses of salt-tolerance mechanisms have been made both in the model plant, *Arabidopsis,* and in crops. However, studies on salt-tolerant genes in halophytes are rare, even though some halophytes have unique mechanisms to cope with high levels of salinity [6–9]. Halophytes are the natural inhabitants of highly saline soils and have evolved to be salt resistant by including efficient control of the uptake and compartmentalization of salt ions, synthesis of organic ‘compatible’ solutes and unique morphological structures, such as succulent leaves, salt glands, and bladders [10]. The genes extracted from the halophytes would be a great resource for studying salt-tolerance mechanisms at molecular level. Salt tolerance mechanism is a multi gene-associated mechanism [11], but many associated genes have not yet been found, due to the complexity of the plant salt tolerance mechanism.

*Salix linearistipularis* (syn. *S. mongolica*) habitats Inner-Mongolia, Heilongjiang, Jilin, Liaoning, Mongolia and (Far-East) Russia. *Salix linearistipularis* is a woody plant that is found in Songnen plain, Heilongjiang, China, that has high salinity and drought salinity at pH more than 9.2 [12]. It plays an important role in maintaining ecological balance and in improving saline soil. There is the genetic information of salt resistance mechanism for the model organisms [13]. However, no noticeable researches in salt resistance mechanism of a non-model plant (such as *S. linearistipularis*) exist. *Salix linearistipularis* is interesting as a non-model organism to study salt resistant features. Model plants and halophytes have been established the salinity stress-related cDNA library and ESTs database. According to DEGs (digital gene expression or differentially expressed gene) technique, significant levels of salt stress-induced DEGs of the model plants are 4–30 % of all-unigenes. Transcriptome analysis enhances and facilitates the understanding of the molecular mechanisms of plant salt tolerance by identifying a large number of salt stress response genes [14–20]. Halophytes maintain the normal metabolism under salt stress by overall stress defending regulation, and the rich response mechanisms to salt stress will be compared with mechanisms in non-salt-stressed plants.

Currently, transcriptome sequencing for rice (*Oryza sativa*) [21], corn (*Zea mays*) [22], and *Arabidopsis thaliana* [23] is applied in the large-scale EST sequencing studies. The DNA sequence information and rich transcriptome sequences of those model plants are well established. These known sequences are used for sequencing, mapping, and assembling genes. The continuous advancement of bioinformatic methods makes transcriptome sequencing for non-model woody plants possible now. However, very few transcriptome studies for non-model woody halophytes are found. Lack of genomic and transcriptome database for non-model woody halophytes becomes main obstacle in the studies of non- model woody halophytes.

In addition, through identification of stress resistance genes and analysis of stress resistance molecular mechanisms for similar or same species in different stress conditions, some stress tolerance plants express stress-related housekeeping genes. Shinozaki [24] compared DEGs for *A. thaliana* and salt mustard (*Thellungiella salsuginea*) using cDNA microarray techniques, and pointed out that the reason why salt tolerance of salt mustard was stronger than that of *A. thaliana* would be the existence of constitutive (housekeeping) expression for salt mustard that matches with salt-inducible expression for *A. thaliana*. Kumari et al. [20] also observed constitutive expressing in salt-tolerance Pokkali, but salt-inducible expression in salt sensitive rice IR64. Brosché et al. [25] found that there was no difference in expression of transcription factors and genes between the control and stress-exposed *Populus* sp.

In this work, we used the seeds of *S. linearistipularis* harvested from saline soil, to identify the salt-resistant genes and to analyze the molecular mechanism. The transcription profiles from both control and salt-stressed *S. linearistipularis* were obtained by using RNA-Seq. The obtained profiles were discussed by comparison with *A. thaliana*, based on gene expression profiling of salt stress-inducible genes, because it is known that *A. thaliana* is a typical model plant [26–28]. All-unigene of control and salt-stressed *S. linearistipularis* samples were classified into 3 categories according to degree of the differences, such as 0–1.5-fold (N-DEGs), 1.5–4.0 and more than 4.0. The genes of three categories were noted by KEGG function, and their pathways were compared with *A. thaliana* to find the salt-resistant KEGG. We were focused on the N-DEGs in *S. linearistipularis* comparable with the DEGs of *A. thaliana* under salt stress condition. Our results provided new insights and the constitutive salt stress mechanisms in *S. linearistipularis*.

## Results and discussion

### Relative electrical conductivity (REC) for salt-treated *S. linearistipularis*

**Fig. 1:**
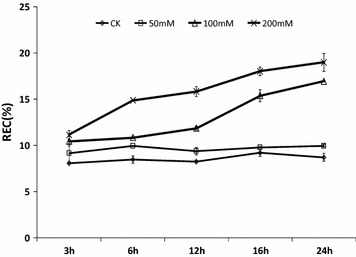
Relative electrical conductivity (REC) for salt-treated *S. linearistipularis*

We have measured the REC of the *S. linearistipularis* in terms of the treated-NaCl concentrations (50, 100, 200 mM) and the treated-time (3, 6, 12, 16, 24 h). In general, REC increased in terms of the exposure to the NaCl concentration and the exposure time. Figure 1 shows that the REC for the 50 mM NaCl treated group did not change significantly with the treated time (*p* > 0.05). For the 100 mM NaCl treated group, the REC increased significantly at 16 h exposure mark as well as for the 200 mM NaCl treated group did at 6 h exposure mark (*p* < 0.05). Because the 100 mM NaCl treated group showed significant increase of the REC after 16 h and also the same significant increase of the REC of the 200 mM NaCl treated group after a 6 h exposure, we decided to use the NaCl treatment conditions (SLH-treated) for 100 mM NaCl for 16 h exposure.

### De novo assembly and quantitative assessment of the Illumina ESTs

The RNA-Seq technique was used to generate two whole-transcriptome profiles of both SLH-control and SLH-treated *S. linearistipularis* groups. The SLH-treated group profile was obtained under the 100 mM NaCl treatment for 16 h. As shown in Table 1, the transcriptome profiles of the two groups contained 27,343,302 and 28,000,000 raw reads, respectively. Adapter reads (>5 % of N reads) and low-quality reads (quality value Q ≤ 10) were eliminated for filtering the valid reads. A total of 25,748,556 and 25,697,734 clean reads were generated from the SLH-control and SLH-treated samples with 98.54 and 98.55 % Q_20_ percentages, respectively. These reads were assembled into 109,567 and 108,481 contigs by the software Trinity. Contig sizes ranged from 200 to 3000 bp. Average contig sizes were 355 bp for the SLH-control group and 352 bp for the SLH-treated group [Additional file 1: Figure S1]. To reduce sequence redundancy, all contigs for SLH-control and SLH-treated groups were further assembled into 60,021 and 60,263 unigenes with size ranging from 200 to 3000 bp. The average size for the SLH-control group and for the SLH-treated group was 684 and 653 bp, respectively [Additional file 2: Figure S2]. To acquire the longest non-redundant sequences possible, unigenes were further assembled into 53,362 non-redundant unigenes, referred to as all-unigenes. All-unigene sizes ranged from 300 to 3000 bp with an average size of 871 bp (Table 2). Size distribution of all-unigene sequences showed that most sequences (23,618; 44.26 %) were no more than 500 bp in length; 23.84 % (12,719) were between 500 and 1000 bp; 29.9 % (15,957) were between 1000 and 3000 bp, while 2 % (1068) were greater than 3000 bp [Additional file 3: Figure S3]. The unigene size distribution showed that shorter fragments were reduced and longer fragments were assembled as a result of further assembly.

**Table 1:** Sequencing statistics of both the salt treated (SLH-treated) and the control (SLH-control) of *S. linearistipularis*

Output statistics	SLH-Control	SLH-Treated
Total raw reads	27,343,302	28,000,000
Total clean reads	25,748,556	25,697,734
Total clean nucleotides (nt)	2,317,370,040	2,312,796,060
Q_20_ percentage	98.54 %	98.55 %
N percentage	0.00 %	0.00 %
GC percentage	45.96 %	45.81 %

Where Q_20_ percentage is proportion of nucleotides with quality value larger than 20 in reads; N percentage is proportion of unknown nucleotides in clean reads. GC percentage is proportion of guanidine and cytosine nucleotides among total nucleotides

**Table 2:** Assembly quality of both SLH-control and SLH-treated *S. linearistipularis*

Assembly quality	SLH-control contig	SLH-treated contig	SLH-control unigene	SLH-treated unigene	ALL unigene
Total number	109,567	108,481	60,263	60,021	53,362
Mean length(nt)	355	352	684	653	871
Total length(nt)	38,859,749	38,194,206	41,232,369	39,191,396	46,456,642
N_50_	645	640	1146	1080	1339
Total consensus sequences			60,263	60,021	53,362
Distinct clusters			23,715	22,999	25,298
Distinct singletons			36,548	37,022	28,064

Where N_50_ is 50 % of the assembled bases were incorporated into sequences with length of N_50_ or longer

All-unigene sequences of *S. linearistipularis* were compared on the basis of similarities to the NCBI non-redundant databases, NR, Swiss-Prot, KEGG, and COG (e value <0.00001) using blastn. These comparisons retrieved proteins with the highest sequence similarity to the *S. linearistipularis* unigenes along with their functional protein annotation information. Among the 53,362 high quality all-unigene sequences, 44,313 (83.04 %) had significant matches to the NR database; 27,399 (51.35 %) to the Swiss-Prot database; 23,885 (44.76 %) to the KEGG database and were assigned to 128 KEGG pathway annotations.

**Fig. 2:**
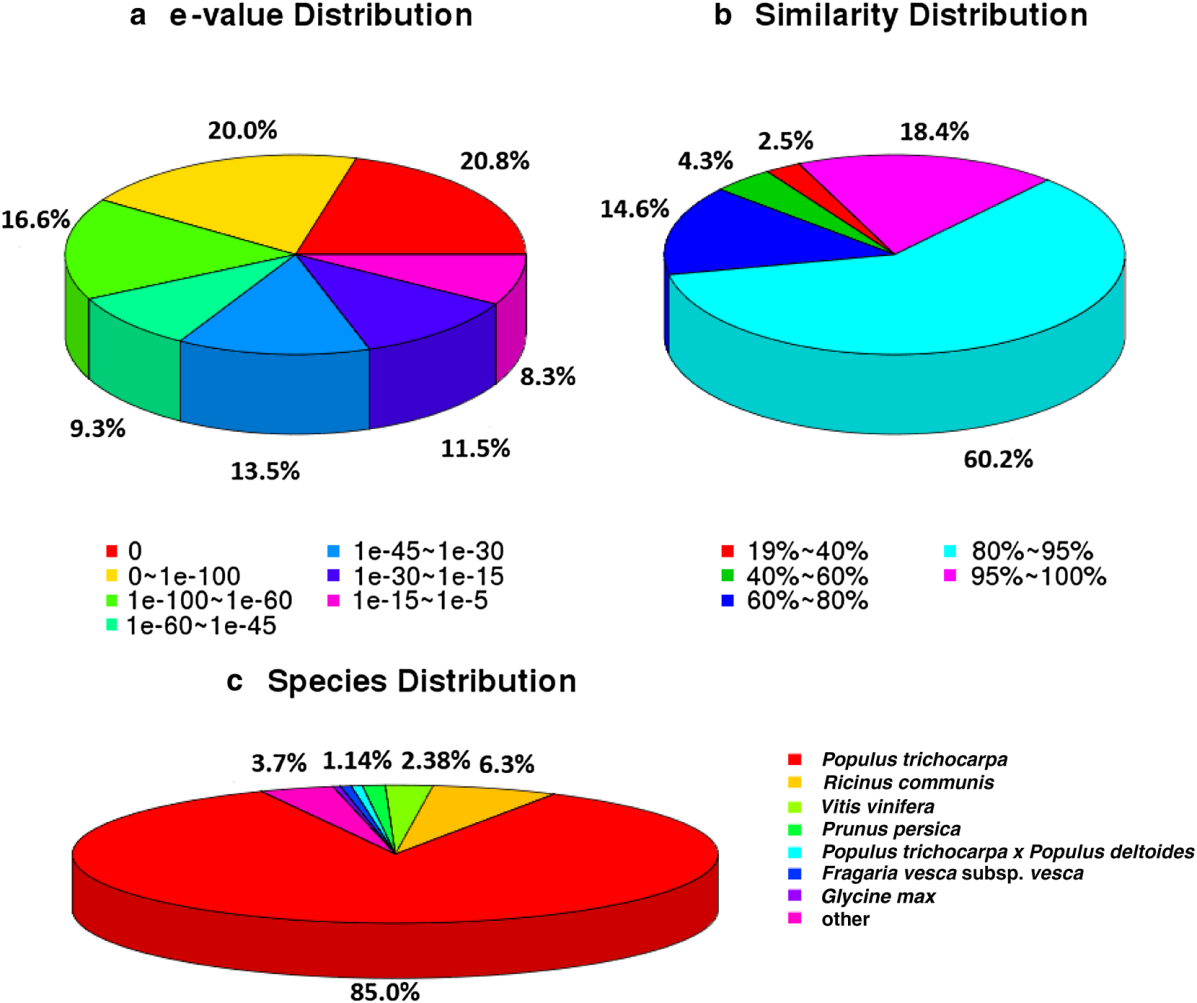
The result of all-unigenes annotation according to NR. **a** e-value distribution, **b** similarity distribution, **c** species distribution

Figure 2a shows e value distribution of the NR database; 8878 reads (20.0 %) at 0–e^−100^, 7341 reads (16.6 %) at e^−100^–e^−60^. Figure 2b shows the identity distribution of the 95–100 % sequence similarity reached 18.4 % of all-unigenes, while the 80–95 % sequence similarity reached 60.2 %. Species distribution (Fig. 2c) shows that 85 % of the unigenes were homologous with *Populus trichocarpa*, minor similarities were followed with castor, grapes, peaches, *P. trichocarpa* × *populus*, strawberries, soybeans and other. For unigenes that were not in the above-described databases, we predicted 471 nucleic acid sequences (sequence 5′ → 3′) and amino acid sequences by using ESTScan.

**Fig. 3:**
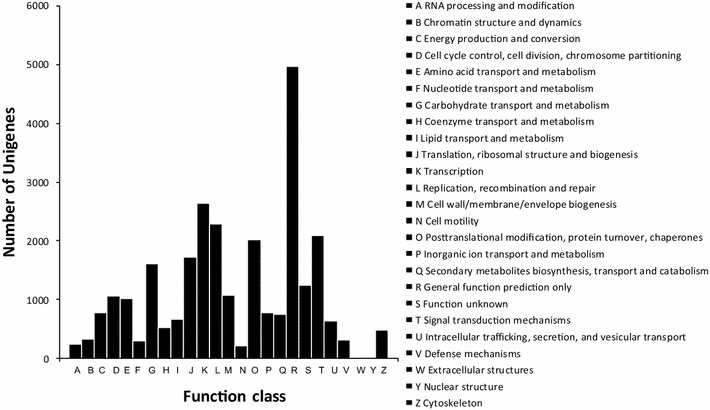
The COG functional classification of all-unigenes. 14,748 (27.64 %) all-unigenes of 53,362 were annotated to COG. All-unigenes were found in 25 functional categories in the COG database

In order to estimate the integrity and effectiveness of the transcriptome data annotation process, we searched for genes that involved COG functional classes. The COG database is a classification of an orthologous gene. Each COG protein is assumed to come from an ancestral protein. By comparing the new sequences and proteins in the COG database, the new sequence can predict and classify possible features. 14,748 unigenes of 53,362 all-unigenes found 25 functional categories in the COG database (Fig. 3). Versatile unigenes were classified into different categories. The category for “General function prediction only” is the most representative class of a tree’s genes (4963 members). Subsequently, categories were followed as shown in Additional file 4: Figure S4.

### GO functional and KEGG pathway analysis

Functions of both salt treated and controlled *S. linearistipularis* genes were predicted by using GO functional annotations to explain how salt treatment affect gene function categories. *Salix linearistipularis* unigenes obtained from the GO functional annotation based on NR annotations; the Blast2GO program was used to get GO annotation of unigenes. Based on sequence similarity, 36,839 unigenes were annotated to 55 function class (Additional file 5: Figure S5). There are 22 biological processes, 17 cellular components, and 16 molecular functions. 23,885 (44.76 %) unigenes were validly matched with the KEGG database, and categorized as 128 known metabolic or signaling pathways (Additional file 6: Figure S6). There were representative pathways as metabolic pathways (5177; 21.67 %), biosynthesis of secondary metabolites (2443; 10.23 %), plant-pathogen interaction (1568; 6.56 %), plant hormone signal transduction (1506; 6.31 %), spliceosome (859; 3.6 %), endocytosis (656; 2.75 %), and protein processing in endoplasmic reticulum (656; 2.75 %). These results demonstrated that salt treatment affected plant metabolic pathways.

### DEG between SLH-treated and SLH-controlled *S. linearistipularis* groups

**Fig. 4:**
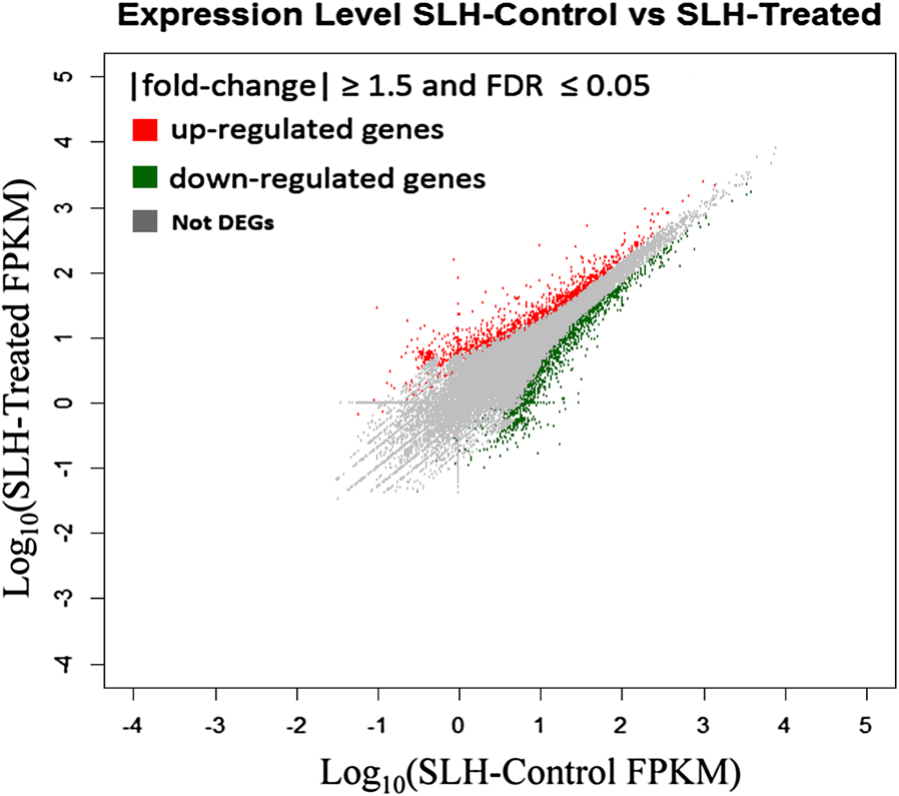
DEGs distribution between SLH-control and SLH-treated *S. linearistipularis* with $\left| {fold - change} \right| \ge 1.5$ and FDR ≤ 0.05. *Red color* is for up-regulated and *green color* is for down-regulated. *Grey color* indicates no significant difference

To identify genes of *S. linearistipularis* with differential expressions under salt conditions, we compared the transcriptome profiles of salt stress treatment (SLH-treated) and control (SLH-control) *S. linearistipularis* groups. Changes in gene expression were calculated with a selected threshold: $\left| {\log_{2}^{Ratio} } \right| \ge 0.584$ (fold − change ≥ 1.5, and FDR ≤ 0.05). 3034 of a total of 53,362 unigenes were DEGs, 1397 up-regulated genes and 1637 down-regulated genes (Fig. 4). All genes were classified by the degree of DEG into three categories: 0–1.5-fold differences in expression levels of unigenes (1768 genes); 1.5–4-fold differences in expression levels (2444 genes); more than 4-fold unigenes (590 genes).

### GO and KEGG pathway analysis of DEGs

**Fig. 5:**
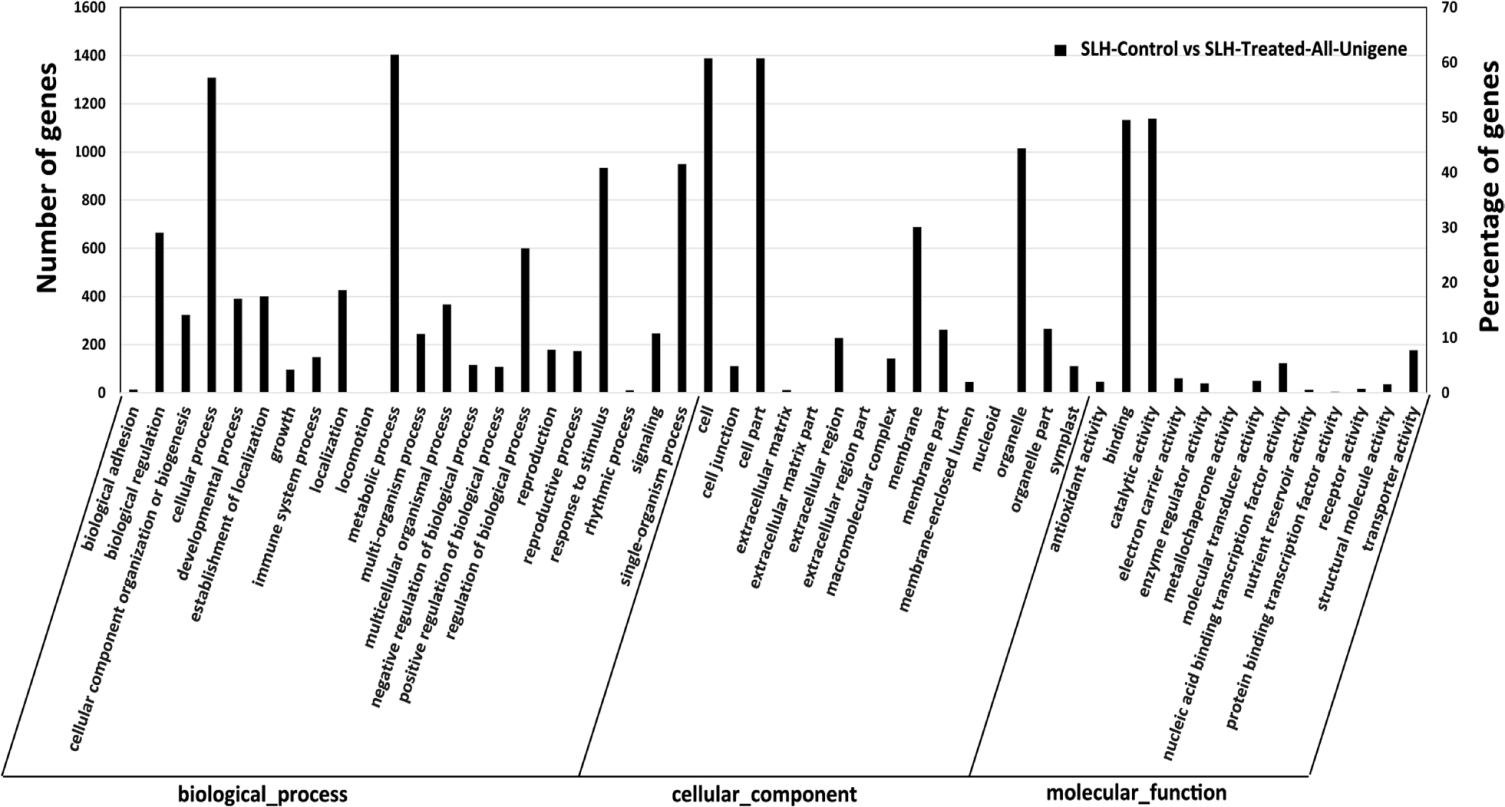
GO Classification of DEGs. GO has 3 ontologies that classified into molecular function, cellular component, and biological process, respectively. The *right side* of *y-axis* indicates the number of DEG,the *left side* indicates the percentage of the total

The GO annotation of *S. linearistipularis* with DEGs under salt conditions ($\left| {fold - change} \right| \ge 1.5$ and FDR ≤ 0.05) identified 50 GO functional classes (Fig. 5). 1855 genes classified to biological process; 1555 genes to the cellular component; and 1765 genes to the molecular function. There were, however, overlapping genes in each ontology annotation. The changes in the biological process indicated that these DEGs were caused from the changes in plant resistance reactions, ion transport, biological redox processes, and the salt resistance mechanism of *S. linearistipularis*. Salt stress-induced molecular function was mainly enriched in oxidoreductase activity, antioxidant activity, peroxidase activity, acting on peroxide as acceptor, active transmembrane transporter activity, and anion transmembrane transporter activity. This explained that antioxidant enzyme activity and transmembrane transport activity in *S. linearistipularis* changed significantly under salt stress. The DEGs in the cellular component was particularly enriched in the extracellular region, cell wall, and central vacuole (corrected *p* value ≤ 0.05).

Similarly, we analyzed the DEGs using the KEGG pathway analysis. Functions of the DEGs between SHL-control and SHL-treated were classified by an interaction analysis, for understanding the biological function pathways at the molecular level. The results showed that the 1103 DEGs were annotated to 116 biological pathways. We were able to conclude that metabolic pathways, biosynthesis of secondary metabolites, plant-pathogen interaction, plant hormone signal transduction, glycerophospholipid metabolism, and endocytosis (*p* ≤ 0.05) were most the representative of effective pathways.

### The integration of KEGG pathway among *S. linearistipularis* and *Arabidopsis* sp.

**Fig. 6:**
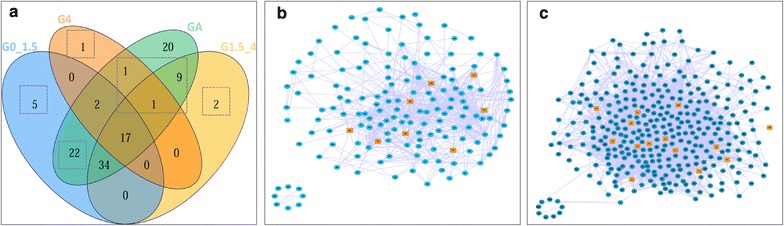
An integrated network is based on the KEGG pathways annotated *by Salix linearistipularis* genes and *Αrabidopsis* sp. DEGs. **a** Venn diagram of the integrated pathways annotated by *S. linearistipularis* and *Arabidopsis* sp. DEGs. GA represents the 107 pathways annotated by the *Arabidopsis* sp. 4799 DEGs. 80 pathways for G0_1.5 (0–1.5-fold DEG) annotated by *S. linearistipularis* unigenes, 63 pathways for G1.5_4 (1.54-fold DEG), 22 pathways for G4 (>4-fold DEG), **b** The integrated networks of 11 overlapped pathways between *S. linearistipularis* and *Arabidopsis* sp., **c** The integrated network of 22 overlapped pathways between *S. linearistipularis* and *Arabidopsis* sp. *Brown dots* represent *S. linearistipularis* genes, corresponding to 38 genes

We found that most of the abiotic stress-induced transcription factors or other transcription factors of *S. linearistipularis* expressed no significant difference between SLH-control and SLH-treated group, but the GO functional annotation showed that they were related to abiotic stress. To observe and describe the salt stress-related transcription, salt stress-induced transcriptome data of *S. linearistipularis* were compared with that of *Arabidopsis* sp. by using the KEGG pathway. As described before, the genes of *S. linearistipularis* were categorized into three categories according to their DEGs levels under salt conditions: 0–1.5 times, 1.5–4 times, more than 4 times. Here, we had these three types of genes annotated to the KEGG pathway: N-DEGs annotated to 80 pathways; 1.5–4-fold DEGs annotated to 63 pathways; above 4-fold DEGs annotated to 22 pathways. 20,488 gene expressions of *Arabidopsis* sp. was downloaded from GEO. We used the SAM algorithm to obtain *Arabidopsis* sp. DEGs under salt stress conditions. We selected thresholds: $\left| {\log_{2}^{Ratio} } \right| \ge 0.584$ and FDR ≤ 0.05 and we received a total of 4799 DEGs in *Arabidopsis* sp. DAVID (Database for Annotation, Visualization and Integrated Discovery) provided the 4799 DEGs a functional annotation. The results show that a total of 107 pathways were annotated by these *Arabidopsis* sp. DEGs. Then, we integrated the pathways annotated by *S. linearistipularis* and *Arabidopsis* sp. DEGs. These compared pathways were drawn into a Venn diagram (Fig. 6a). From Fig. 6a, we found that 22 pathways annotated by *Arabidopsis* sp. DEG’s overlapped with those annotated by the *S. linearistipularis* N-DEG levels at 0–1.5 times, and 11 pathways did at more than 1.5 times. Then XML files were downloaded from the KEGG database corresponding to 22 and 11 pathways. The XML package of R software was used to obtain the genes and their relations to the pathways. Furthermore, these genes and their relations were mapped into a network by Cytoscape, a network mapping software (Fig. 6b). Figure 6b shows the integrated network based on the 11 pathways, which contains 158 points and 791 sides. The yellow dots represent *S. linearistipularis* genes, corresponding to 12 genes. These genes and their expression levels are listed in Table 3. Figure 6c shows the integrated network based on the 22 pathways, which contains 343 points and 3059 sides. The brown dots represent *S. linearistipularis* genes, corresponding to 38 genes. These genes and their expression levels are listed in Table 4.

**Table 3:** Eleven overlapped *S. linearistipularis* pathways with *Arabidopsis* sp.

Pathway	GeneID	Gene annotation	Log_2_(SLH-Treated_FPKM/SLH-Control_FPKM)	FDR
Ubiquinone and other terpenoid-quinone biosynthesis	CL9057.Contig1_All	1,4-Dihydroxy-2-naphthoyl-CoA synthase	1.5475	1.39 × 10^−03^
Isoquinoline alkaloid biosynthesis	Unigene2060_All	aminotransferase family protein	0.6377	1.07 × 10^−07^
Nicotinate and nicotinamide metabolism	Unigene24510_All	SufE-like protein	1.1473	4.24 × 10^−03^
Non-homologous end-joining	Unigene15563_All	DNA repair protein RAD50	−1.4166	8.52 × 10^−04^
Homologous recombination	Unigene15563_All		−1.4166	8.52 × 10^−04^
Ascorbate and aldarate metabolism	Unigene9728_All	Inositol oxygenase 1	0.9425	2.06 × 10^−10^
	CL5877.Contig2_All	Inositol oxygenase 2	0.6185	4.80 × 10^−05^
Phosphatidylinositol signaling system	CL1261.Contig1_All	predicted protein	−0.713	6.60 × 10^−04^
	CL8598.Contig2_All	predicted protein	−2.4993	2.96 × 10^−03^
Inositol phosphate metabolism	CL5625.Contig1_All	predicted protein	−1.3322	7.07 × 10^−04^
Fructose and mannose metabolism	CL2465.Contig5_All	predicted protein	0.8893	9.94 × 10^−06^
Biosynthesis of unsaturated fatty acids	CL4746.Contig1_All	Acyl-coenzyme A oxidase 3	0.6963	6.79 × 10^−29^
Pentose and glucuronateinterconversions	CL9179.Contig1_All	putativepectatelyase 2	−2.1229	6.99 × 10^−07^

**Table 4:** Twenty-two overlapped pathways that have no DEG level in *S. linearistipularis* but DEG level at more than 1.5-fold in *Arabidopsis* sp.

Pathway	GeneID	Gene annotation	SLH-control	SLH-treated	Log_2_ (treated/control)	FDR
Proteasome	CL3345.Contig2	26S protease regulatory subunit	59.8522	72.6467	0.2795	0.000426
	CL7794.Contig1	26S protease regulatory subunit	99.0566	112.3448	0.1816	0.007457
	CL2535.Contig2	predicted protein	39.0475	46.8721	0.2635	0.00905
	CL7718.Contig2	Proteasome subunit beta type-2	47.5126	70.1633	0.5624	8.89 × 10^−08^
	CL3715.Contig2	26S proteasome non-ATPase regulatory subunit	16.6796	22.5668	0.4361	0.00345
	CL7840.Contig2	26S proteasome non-ATPase regulatory subunit	74.8551	88.6333	0.2437	0.00946
Protein export	Unigene11088	Cell division protein FtsY homolog	17.7825	12.6174	−0.495	0.007987
	CL7191.Contig1	Signal recognition particle 54 kDaprotein	22.6598	17.5094	−0.372	0.004058
Beta-Alanine metabolism	Unigene18351	Isovaleryl-CoA dehydrogenase 1	80.617	92.5123	0.1986	9.1 × 10^−05^
	Unigene2254	N-carbamoylputrescineamidase	55.0139	68.1933	0.3098	0.000129
RNA degradation	Unigene13605	Enhancer of mRNA-decapping protein	30.479	24.9409	−0.2893	5.27 × 10^−05^
	CL7185.Contig2	predicted protein	24.2334	16.6728	−0.5395	0.001459
Pantothenate and CoA biosynthesis	Unigene2254	N-carbamoylputrescineamidase	55.0139	68.1933	0.3098	0.000129
	CL5521.Contig2	Ketol-acid reductoisomerase	68.8746	58.4374	−0.2371	0.000509
Metabolism of xenobiotics by cytochrome P450	CL1909.Contig1	Alcohol dehydrogenase class-3	125.939	177.1319	0.4921	9.59 × 10^−30^
Lysine biosynthesis	CL6463.Contig1	Alpha-aminoadipicsemialdehyde synthase	27.7034	38.3763	0.4702	1.67 × 10^−11^
	Unigene8491	Diaminopimelate decarboxylase 2	53.4166	62.6334	0.2296	0.005103
	CL3845.Contig2	Diaminopimelateepimerase	50.2853	60.5122	0.2671	0.003409
Lysine degradation	CL6463.Contig1	Alpha-aminoadipicsemialdehyde synthase	27.7034	38.3763	0.4702	1.67 × 10^−11^
Arginine and proline metabolism	CL8463.Contig2	Glutamate dehydrogenase 1	44.4049	56.5985	0.35	0.001105
Porphyrin and chlorophyll metabolism	CL646.Contig5	Glutamyl-tRNAreductase 1	13.3057	9.1201	−0.5449	0.001246
Histidine metabolism	Unigene9767	Imidazole glycerol phosphate synthase hisHF	32.2903	25.6821	−0.3303	0.001729
One carbon pool by folate	CL2146.Contig1	Serine hydroxymethyltransferase	70.804	86.6454	0.2913	8.51 × 10^−07^
	CL6648.Contig1	Serine hydroxymethyltransferase	180.7253	259.5866	0.5224	7.49 × 10^−55^
	Unigene11248	Serine hydroxymethyltransferase	40.4825	49.1695	0.2805	0.00285
SNARE interactions in vesicular transport	CL1271.Contig1	VAMP-like protein	33.3667	42.716	0.3564	0.009107
	CL5949.Contig2	Vesicle transport v-SNARE	27.6954	40.8223	0.5597	0.000616
Caffeine metabolism	Unigene483	Xanthine dehydrogenase	23.2954	29.0532	0.3187	1.03 × 10^−05^
Tropane, piperidine and pyridine alkaloid biosynthesis	CL140.Contig1	Phenylalanine ammonia-lyase	153.4218	229.943	0.5838	5.89 × 10^−08^
	CL140.Contig3	Phenylalanine ammonia-lyase	386.8141	418.3274	0.113	6.97 × 10^−06^
Glycine, serine and threonine metabolism	Unigene11172	D-3-phosphoglycerate dehydrogenase	68.4341	83.2202	0.2822	2.51 × 10^−06^
	Unigene11248	Serine hydroxymethyltransferase	40.4825	49.1695	0.2805	0.00285
	CL2146.Contig1	Serine hydroxymethyltransferase	70.804	86.6454	0.2913	8.51 × 10^−07^
	Unigene18435	Threonine dehydratase biosynthetic	97.1851	130.6801	0.4272	3.98 × 10^−24^
	CL6648.Contig1	Serine hydroxymethyltransferase	180.7253	259.5866	0.5224	7.49 × 10^−55^
Spliceosome	CL1782.Contig1	predicted protein	27.796	19.9794	−0.4764	1.03 × 10^−17^
	Unigene3088	predicted protein	79.8566	95.1218	0.2524	0.003244
	Unigene15805	Protein pleiotropic regulatory locus	40.8867	31.9293	−0.3568	8.01 × 10^−05^
	CL1939.Contig3	heat shock 70 kDa	41.4488	52.1506	0.3314	9.96 × 10^−06^
	Unigene5315	predicted protein	34.3227	47.38	0.4651	0.001735
Fatty acid biosynthesis	CL1034.Contig2	Acetyl-CoA carboxylase 1	19.096	16.5839	−0.2035	0.008148
DNA replication	Unigene2892	Proliferating cell nuclear antigen	26.9745	35.3695	0.3909	0.006219
Base excision repair	Unigene2892		26.9745	35.3695	0.3909	0.006219
Mismatch repair	Unigene2892		26.9745	35.3695	0.3909	0.006219
Natural killer cell mediated cytotoxicity	CL3893.Contig3	Mitogen-activated protein kinase 3	180.9353	136.4617	−0.407	2.54 × 10^−17^

Figure 6 showed that first, second, and fifth pathways of the *S. linearistipularis* DEGs for >4, 4–1.5 and 0–1.5 times (NDEGs), respectively, did not overlap with *Arabidopsis* sp. The first pathway was the RNA polymerase glycolysis/gluconeogenesis; the second was the fatty acid elongation in mitochondria and polyketide sugar unit biosynthesis; and the fifth pathway was the biosynthesis of alkaloids derived from ornithine lysine and nicotinic acid, glycine serine and threonine metabolism, alanine aspartate and glutamate metabolism, valine leucine and isoleucine biosynthesis, and sphingolipid metabolism.

Table 3 lists the overlapping KEGG annotated pathways for *Arabidopsis* sp. DEGs and more than 1.5 times DEGs of *S. linearistipularis*. The primary pathways were: CL9057.Contig1 (encoding 1,4-dihydroxy-2-naphthoyl-CoA synthase), unigene2060 (encoding AMT), unigene9728, CL5877.Contig2 (encoding *myo*-inositol oxygenase), and CL4746.Contig1 (encoding fatty acyl coenzyme A dioxygenase); among them,CL5877.Contig2was previously reported as the salt stress-related genes [29].

Table 4 lists the overlapping KEGG annotated pathways in both the DGEs of *Arabidopsis* sp. and 0–1.5 times N-DEGs of *S. linearistipularis*. The FPKM values of both the control and treatment groups were generally high. Five of 38 unigenes were at FPKM > 100 and 17 were at FPKM > 50. Other FPKM values were low, as CL1034.Contig2, CL646.Contig5, unigene11088, and CL3715.Contig2 measured at 19.096, 13.3057, 17.7825, and 16.6796, respectively. Homologous unigenes of *S. linearistipularis* to *Arabidopsis* sp., CL2146.Contig1, CL6648.contig1, unigene11248 (encoding SHMT), CL5949.Contig2 (encoding v-SNARE), CL1939.Contig3 (encoding Hsp70), CL3893.Contig3 (encoding MPK3), CL3845.Contig2 (encoding glutamate dehydrogenase), and others were up regulated in salt-treated *Arabidopsis* sp. However, there was no significant difference between SLH-control and SLH-treated *S. linearistipularis*. These observations show that various genes induced by salt stress in *Arabidopsis* sp. are overexpressed in unstressed conditions in *S. linearistipularis*. This suggested that *S. linearistipularis*, like other woody plants, grew for a long time under saline and or drought soil conditions and that stress-inducible signaling pathways are constitutive and active in *S. linearistipularis* even under normal growth conditions without salt stress.

### Validation of DEGs and N-DEGs by quantitative RT-PCR

**Fig. 7:**
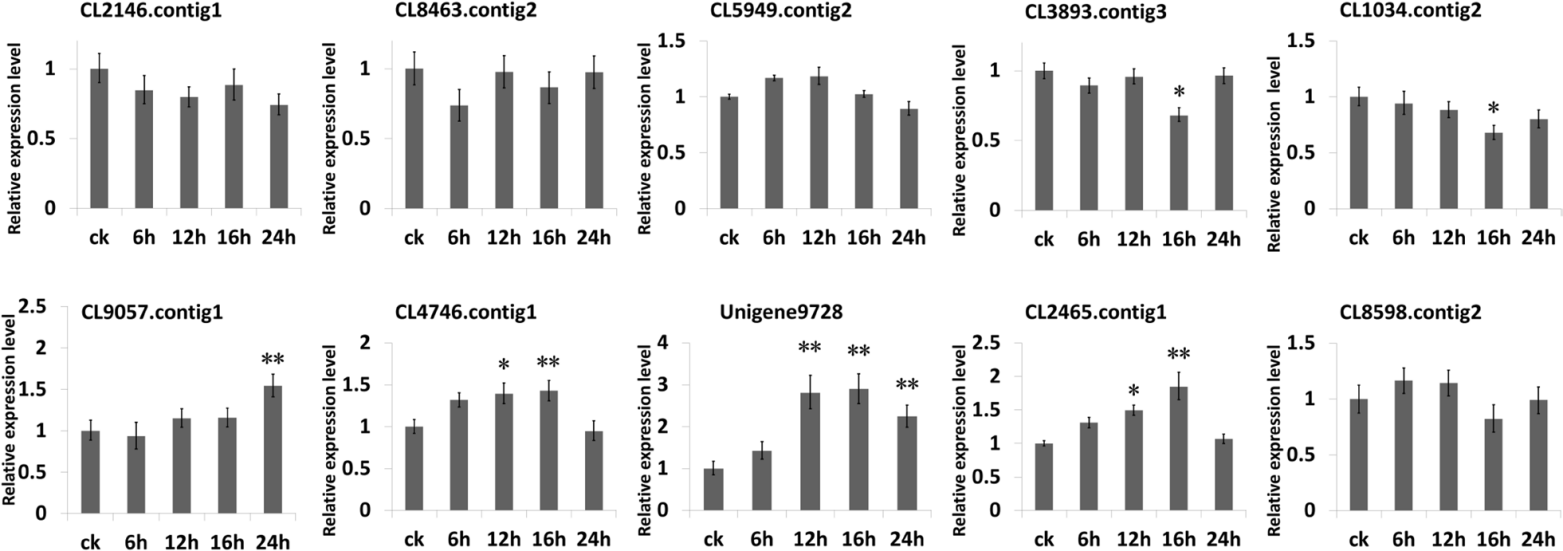
The quantitative real-time PCR analysis of selected unigenes. The *upper column* was 5 selected unigenes of 22 pathways (no DEG level in *S. linearistipularis* but DEG level at more than 1.5-fold in *Arabidopsis* sp.*),* and the *lower column* was 5 selected unigenes of 11 pathways (that has DEG levels at more than 1.5-fold). ^*^ and ^**^ indicate significant differences at 0.05 and 0.01 probability levels, respectively

To validate the sequencing results, a quantitative RT-PCR test was performed, from which five genes were selected: CL2146.Contig1, CL8463.Contig2, CL3893.Contig3, CL5949.Contig2, and the CL1034.contig2; among which the 22 intersected pathways of *S. linearistipularis* were validated. Also, as shown in Fig. 7, additional five genes (CL9057.Contig1, CL4746.Contig1, unigene9728, CL2465.Contig5 and CL8598.contig2) were validated among 11 intersected pathways of *S. linearistipularis*.

*CL2146.Contig1* is a homologous gene of *Iopulus tremuloides* serine hydroxymethyltransferase (SHMT) and a homologous gene of *A. thaliana* SHM1. SHM1 is involved in photorespiration and salt tolerance of *Arabidopsis* sp. [30]. UBP16 (UBP16 is a ubiquitin-specific protease; the activity of this enzyme is required for salt tolerance) is involved in salt tolerance of *Arabidopsis* sp. by modulating the sodium transport activity and repressing cell death at least partially through modulating the SMH1stability and activity [30]. Quantitative RT-PCR results showed that no significant differences appeared between SLH-treated and SLH-control at all time periods. However, the CL2146.Contig1 gene was already at a higher expression in the SLH-control than in the SLH-treated (*p* < 0.01).

*CL8463.Contig2* is a homologous gene of *P. trichocarpa* Glutamate Dehydrogenase 1 (GDH1) family protein and a homologous gene of *A. thaliana* GDH1. GDHs catalyze the reduction of the α-ketoglutarate plus ammonia (ammonification), and catalyze an oxidative deamination of glutamate (ammonification), which is widely presented in plant tissues [31]. Studies showed that when salt stress is increased, ammoniated activity of salt-tolerance rice GDH proteins increase and the ammoniated activity of salt-stress sensitivity of rice GDH proteins weakened. Our quantitative RT-PCR results showed no significant difference in expression between the SLH-treated and SLH-control (*p* > 0.01).

*CL5949.Contig2* is a homologous gene of vesicle transport v-SNARE in *Vitis vinifera*. The function of the tonoplast-specific v-SNAREs (AtVAMP71/AtVAMP7C) is for responding water deficiency in the plant [32]. The AtVAMP71 complex plays an important role in the proper ROS localization on *A. thaliana* guard cells, regulating stomatal closure after ABA treatment [32]. As shown in Fig. 7, quantitative RT-PCR showed that the CL5949.Contig2 expression increased by 40 % from 6–12 h salt treatment and decreased at 16–24 h salt treatment.

*CL3893.Contig3* is a homologous gene of mitogen-activated protein kinase 3 (MPK3) in *A. thaliana*. The MKK4 plays an important role in plants under osmotic stress, which is involved in the osmotic-stress response via its regulation of MPK3 activity [33]. Our quantitative RT-PCR of CL3893.Contig3 revealed no significant differences between the SLH-control and SLH-treated at various time periods (*p* > 0.01).

*CL1034.Contig2* is a homologous gene of Acetyl-CoA Carboxylase 1 in *A. thaliana*, its expression under salt exposure was lower than that of the control group.

*CL9057.Contig1* is a homologous gene of MENB in *A. thaliana*.

*CL4746.Contig1* is a homologous gene of Acyl-coenzyme A oxidase (ACOX3) in *A. thaliana*. CL9057.Contig1 expression began to increase from the 12 h salt treatment mark, whereas CL4746.Contig1 showed increased expression after 6 h of salt treatment and began to decline after 24 h of salt treatment.

*Unigene9728* is a homologous gene of MIOX1 *(myo*-inositol oxygenase) in *A. thaliana*. MIOX is one of the key enzymes in plant ascorbic acid biosynthetic reactions [34]. It is found in the ascorbic acid synthesis pathway of *Arabidopsis* sp. Starting with the *myo*-inositol, an inositol combined enzyme is a key enzyme for this biosynthetic pathway. Ascorbic acid in plants not only regulate growth and development, but also act as an antioxidant in scavenging superoxide radicals and ROS, such as singlet oxygen and hydrogen peroxide substances and reducing reactive oxygen damage caused to cells [29]. At low temperatures, drought and high salt stress conditions, ascorbic acid functions to protect plants. RT-PCR results showed that Unigene9728 expression increased between 6 and 16 h of salt treatment, but decreased after 24 h.

*CL2465* is a homologous gene of fructokinase-4 in *A. thaliana*. Its expression increased gradually up to 16 h of salt stress exposure, but further exposures caused decreasing.

*CL8598.Contig2* is a predicted protein. Its expression increased ~20 % at the early exposure time but declined after16 h of exposure.

Overall, our quantitative RT-PCR analyses and transcriptome sequencing were consistent with each other.

## Conclusions

1. The genomic sequence and functions of *S. linearistipularis* were noted by using transcriptome data in this study. This paper provided for the first time the genetic information of *S. linearistipularis* with gene expressions and their functions. The analysis results showed that 85 % of the genes overlapped with the Comospore genome, and the 85 % of genes that overlapped with other species including *Ricinus communis*, *Vitis vinifera*, *Prunus persica*, *P. trichocarpa*, *P. deltoids*, and others. This suggests that the genome of *S. linearistipularis* contains a different gene and the DEGs contributed to the adaptation to the salt stress. Of the 53,362 all-unigenes, the 3134 DEGs ($\left| {\log_{2}^{Ratio} } \right| \ge 0.584$, FDR ≤0.05), included 1397 up-regulated genes and 1637 down-regulated genes. The cluster analyses revealed 2199 genes were clustered in 50 GO terms, and 1103 genes were clustered in 116 biological pathways.
2. To find the DEGs of the more than 1.5-folds in *S. linearistipularis*, the pathway genes were integrated with those pathways related to *Arabidopsis* sp. Eleven pathways from the more than 1.5-fold categories were the same as with *Arabidopsis* sp. pathways.
3. To find the N-DEGs at the 0–1.5-folds in *S. linearistipularis*, the pathway genes were integrated with those pathways related to *Arabidopsis* sp. The 22 pathways from N-DEGs overlapped with *Arabidopsis* sp. pathways.
4. Based on the expression profiling, it was demonstrated that various genes induced by salt stress in *Arabidopsis* sp. were overexpressed in unstressed conditions in *S. linearistipularis*. This suggests that stress-inducible signaling pathways were constitutive and active in *S. linearistipularis* even under normal growth conditions without salt stress.

## Methods

### Plant samples and culture conditions

*Salix linearistipularis* seeds were harvested from saline soil of Anda experimental site (Anda, Heilongjiang, China) in May. The seeds were cultured in 0.8 % agar and 2.5 % sucrose 1/2MS solid medium at 22 ± 1 °C with a photoperiod of 12 h light and 12 h dark until five true leaf growth. The salt treatment conditions of *S. linearistipularis* seedlings (SLH-treated group) were 0, 3, 6, 12, 16 and 24 h in 100 mM NaCl added at 1/2MS medium, and compared with no NaCl-treated control (SLH-control group).

### Conductivity measurements

After *S. linearistipularis* seedlings were treated to 3, 6, 12, 16 and 24 h with an additional 50, 100, 200 mM NaCl in 1/2MS solid medium, REC was measured and compared with the SLH-control group. The conductivities were measured according to Yao et al. [35].

### RNA extraction and quality assessment

The CTAB method was used to extract total RNAs of *S. linearistipularis*. Every sample was selected separately for each treatment consistent with the size of six *S. linearistipularis* seedlings. Using isopropanol precipitation, the DNA was digested with DNaseI to obtain total RNAs of *S. linearistipularis*. The RNA samples were used to build a database that met with the quality requirements using NanoDrop (Thermo Scientific, USA). Additionally, using the Agilent 2100 (Agilent, USA) or Caliper Lab Chip GX (Caliper Lifescience Inc., USA) detection, the database of the total RNA samples was refined to meet the requirements. The 28S/18S concentration ratio and RIN value of SLH-treated group were 1474 ng µl^−1^, 1.3 and 7.7 ng µl^−1^, respectively, while those of the SLH-control group were 1870, 1.6 and 8.6 ng µl^−1^, respectively.

### Library construction and sequencing of cDNA

An alternated CTAB method was used for the total RNA extraction of the *S. linearistipularis*. After the DNaseI treatment, magnetic beads with Oligo (dT) were used to isolate the mRNA (for eukaryotes) or by removing rRNAs from the total RNA (for prokaryotes). By mixing the fragmentation buffer in the Thermomixer^®^ (Eppendorf AG, Germany), the mRNA was segmented into short fragments. Then cDNA was synthesized using the mRNA fragments as templates. Double stranded cDNA was then synthesized. Short fragments were purified and resolved with EB buffer for end reparation and single nucleotide A addition. Subsequently, the short fragments were connected with adapters. Suitable fragments were then selected for the PCR amplification as templates. During the QC steps, the Agilent 2100 Bioanaylzer (Agilent, USA) and the ABI StepOnePlus Real-Time PCR System (ABI, USA) were used in the quantification of the sample library. Finally, the library was sequenced using the Illumina HiSeq™ 2000 (Illumina, USA).

### Transcriptome de novo assembly

Image data output from the sequencing machine was transformed by base calling into sequence data in fastq format, which was called raw data or raw reads. Raw reads produced from sequencing machines contained dirty reads (which contained adapters, unknown or low quality bases), which they were discarded under the following criteria: (1) reads with adaptors, (2) reads with unknown nucleotides larger than 5 %, (3) low quality reads in which the percentage of low quality bases (base quality ≤ 10) was more than 20 %. Transcriptome de novo assembly was carried out with the short reads assembling program—Trinity [36]. The result sequences of Trinity were called unigenes. When multiple samples from the same species were sequenced, unigenes from each sample assembly could be taken for further processing of sequence splicing and redundancy removing, with sequence clustering software (Illumina Inc., USA) to acquire non-redundant unigenes as long as possible.

Gene family clustering was then grouped into two classes. One cluster was given the ‘CL’ prefix and id number. Each cluster was grouped by unigenes with >70 % similarity. The others were singletons, with the prefix unigene. In the final step, blastx alignment (e value < 0.00001) of unigenes with the protein databases of NR, Swiss-Prot, KEGG and COG were performed. The best aligning results were used for sequence direction of the unigenes. If there was a conflict between the different databases, we followed the priority order of NR, Swiss-Prot, KEGG and COG for build sequence direction of unigenes. When a unigene was aligned to none of the above databases, the ESTScan software (http://myhits.isb-sib.ch/cgi-bin/estscan) [37] was used to build its sequence direction.

### Coding sequences CDS

Unigenes were initially aligned by blastx (e value < 0.00001) based on the priority order of NR, Swiss-Prot, KEGG and COG. The alignments were terminated when all alignments were finished. Proteins with the highest ranks in blast results were taken as CDs of unigenes, and then CDs were translated into amino acid sequences with the standard codon table. In this way, both the nucleotide sequences (5′→3′) and amino acid sequences of the unigene-coding region were acquired. For unigenes that could not be aligned to any database, we scanned them with the ESTScan in order to obtain the nucleotide sequence (5′→3′) direction and amino sequence of the predicted coding region.

### The functional annotation of unigene, GO category and KEGG pathway analysis

With NR annotation, we used the Blast2GO program to get the GO annotation of unigenes. Information of functional annotations provided protein functional annotations, COG functional annotations and GO functional annotations of the unigenes. The obtained unigene sequences were retrieved with proteins that have the highest sequence similarity with the given unigenes along with their protein functional annotations from the databases, NR and Swiss-Prot. Every protein in the COG annotation was assumed to evolve from an ancestor protein, and the whole database was built as coding proteins with a complete genome as well as a system evolution relationship of bacteria, algae and eukaryotic organisms. With the KEGG annotation we built a pathway annotation of unigenes. Analysis of all-unigene annotations revealed information on the amount of expression and function in each sample.

### The calculation of unigene expression

The FPKM method (Fragments Per kb per Million fragments) was used to calculate the unigene expression; FPKM = 109C/NL, where C is number of fragments that uniquely aligned to a unigene, N is total number of fragments that uniquely aligned to all-unigenes, and L is the base number in the cording sequence (CDs) of unigene. In our analysis, the DEGs were classified into three groups (0–1.5 times, 1.5–4 time and >4 times) and compared to the KEGG functional annotation of these groups for the DEGs FDR ≤ 0.05 with $\left| {fold - change} \right| \ge 1.5$. After obtaining the DEGs, we conducted a GO (corrected *p* value ≤ 0.05) functional analysis and a KEGG Pathway (Q value ≤ 0.05) analysis.

### Gene expression profiles of salt stressed *Arabidopsis*

Gene expression data, GSE5623 and GSE5620, of the salt-stressed *Arabidopsis* sp. was downloaded from the GEO database. GSE5623 was from the salt-treated samples for 0.5, 1, 3, 6, 12, 24 h, and GSE5620 was from the corresponding control group. Using the SAM method, the difference of the *Arabidopsis* sp. gene expression between the salt-treated group and control group was calculated with a selected threshold $\left| {fold - change} \right| \ge 1.5$ times, FDR ≤ 0.05. Then, the obtained DEGs of *Arabidopsis* sp. genes were used to do a functional annotation, gene annotation, and a KEGG pathway by using DAVID [38].

### KEGG pathway annotation of DEG for *S. linearistipularis* and *Arabidopsis* sp

In the previous data analysis and processing, the KEGG pathways of the three classified DEG groups in both *Arabidopsis* sp. and *S. linearistipularis* were obtained. Using an R (http://www.r-project.org) grid and a Venn diagram package [39], a Venn diagram of these KEGG pathways was built. The Venn diagram visualized the overlapped of DEGs between *Arabidopsis* sp. and *S. linearistipularis*. The overlapping pathways were downloaded onto an XML file from the KEGG database, and then using R in the XML file, gene correlations were extracted from the corresponding KEGG pathway. These extracted correlations were used to build an integrated network by Cytoscape (http://www.cytoscape.org/index.html) [40].

### Quantitative real-time PCR

RNA was extracted from *S. linearistipularis* that incubated at different exposure times (i.e. 6, 12, 16 and 24 h) in 100 mM Nacl 1/2MS solid medium. No salt treated samples were used as a control. Synthetic cDNA was synthesized using a reverse transcription kit (Takara-Bio, Dalian, China). The primers for RT-PCR and quantitative RT-PCR were designed by using the Primer 5.0 software (Primer Biosoft, USA) [Additional file 7: Figure S7]. Actin was used to make a reference gene [41]. Quantitative RT-PCR analysis was performed using the MX3000P quantitative fluorescence detection system (Genetimes Technology Inc., China), a 20 μl reaction system and SYBR Premix (Agilent Technologies, USA). The conditions of quantitative RT-PCR analysis were 95 °C for 2 min, and followed by 40 cycles at 95 °C for 30 s, 60 °C for 30 s and 72 °C for 30 s.

## Additional files

10.1186/s40709-016-0038-7 The length distribution of contigs in control and treated *S. linearistipularis.*

10.1186/s40709-016-0038-7 The length distribution of unigene in control and treated *S. linearistipularis.*

10.1186/s40709-016-0038-7 Length distribution of all-unigene extracted from *S. linearistipularis.*

10.1186/s40709-016-0038-7 Category of predicted *S. linearistipularis* proteins among functional groups.
10.1186/s40709-016-0038-7 Go annotation of *S. linearistipularis* all-unigenes.
10.1186/s40709-016-0038-7 Pathway annotation of *S. linearistipularis* all-unigenes.
10.1186/s40709-016-0038-7 qRT-PCR Primer design.

## Abbreviations

COG: clusters of orthologous groups
KEGG: Kyoto encyclopedia of genes and genomes
DEG: differentially expressed genes
ROS: reactive oxygen species
CTAB: hexadecyltrimethylammonium bromide
GO: gene ontology
FPKM: fragments per kb per million fragments
FDR: false discovery rate
CDS: coding sequence
EST: expressed sequence tags
NCBI: National Center of Biotechnology Information
GEO: gene expression omnibus
SHMT: serinehydromethyl-transferase
GDH: glutamate dehydrogenase
V-SNARE: vesicle soluble *N*-ethylmaleimide-sensitive factor attachment protein receptors
MAPK: mitogen-activated protein kinase
