# Supplementary figures and images for: NaCl stress-induced transcriptomics analysis of Salix linearistipularis (syn. Salix mongolica)
Source: J Biol Res (Thessalon). 2016 Feb 29;23:1. doi: 10.1186/s40709-016-0038-7 (PMC4772304; doi:10.1186/s40709-016-0038-7)

## Slide 1
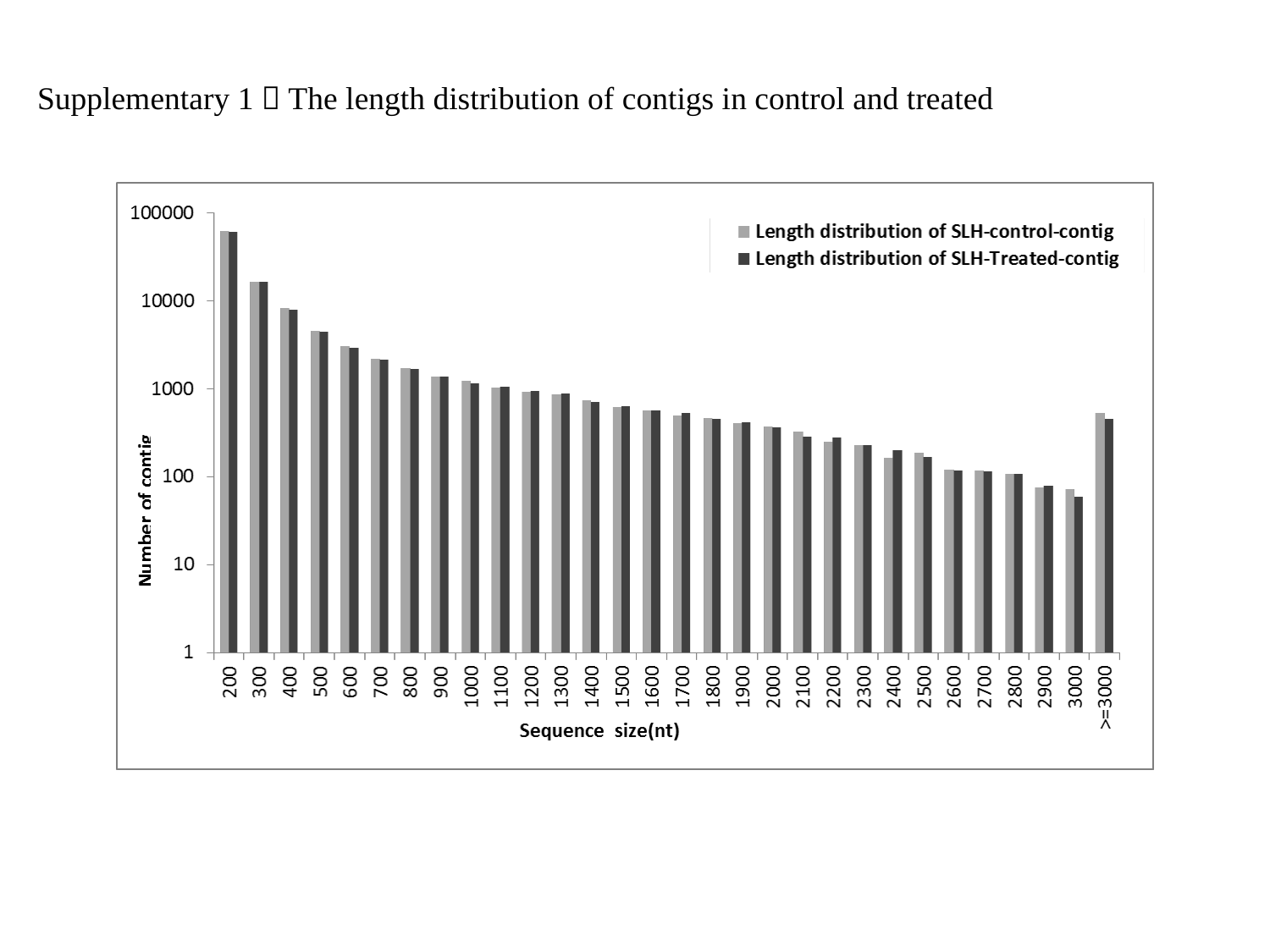

Supplementary 1：The length distribution of contigs in control and treated

Supplement: Supplementary file 1 — 10.1186/s40709-016-0038-7 The length distribution of contigs in control and treated S. linearistipularis. [file 40709_2016_38_MOESM1_ESM.pptx]

## Slide 1
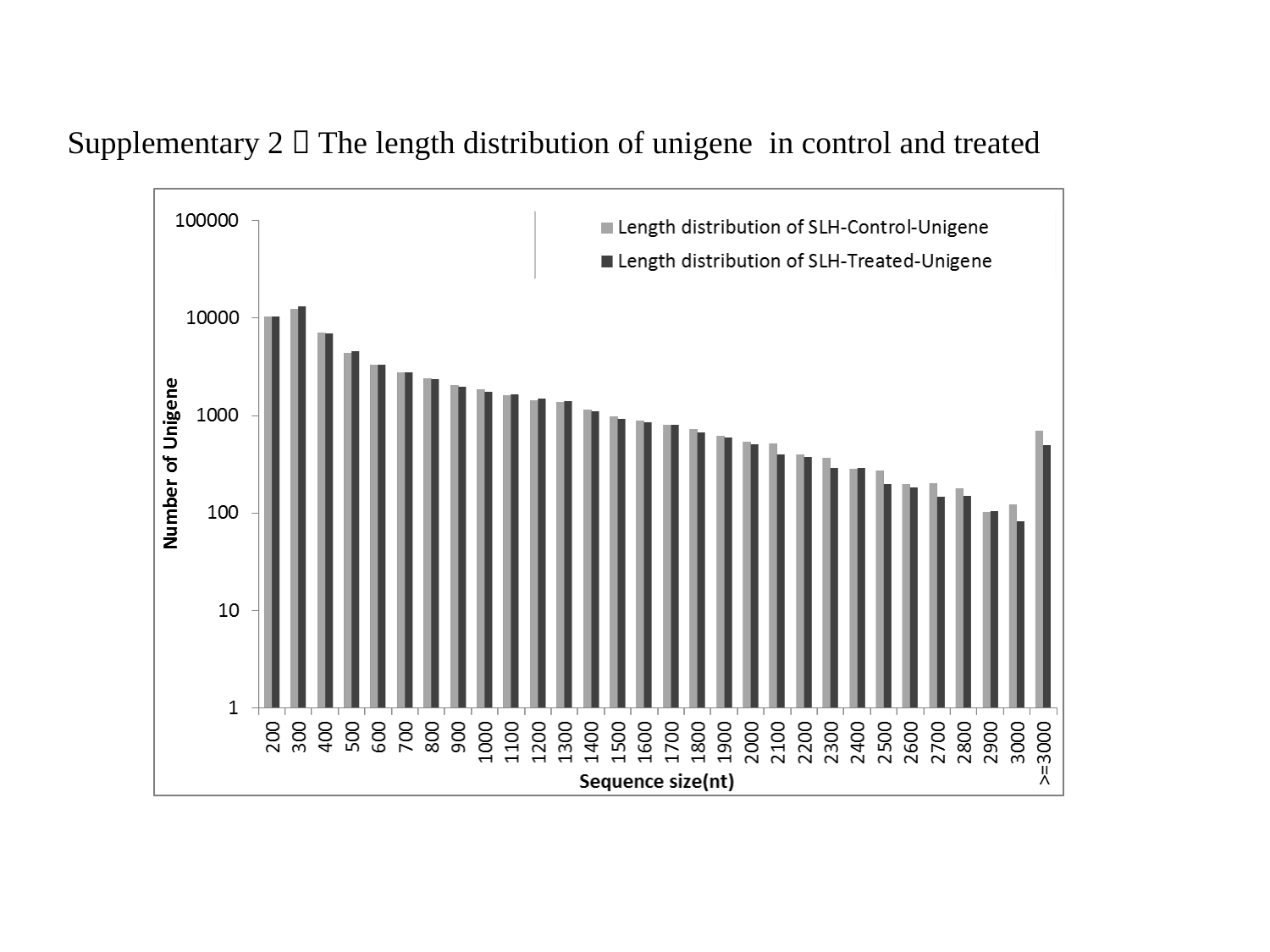

Supplementary 2：The length distribution of unigene in control and treated

Supplement: Supplementary file 2 — 10.1186/s40709-016-0038-7 The length distribution of unigene in control and treated S. linearistipularis. [file 40709_2016_38_MOESM2_ESM.pptx]

## Slide 1
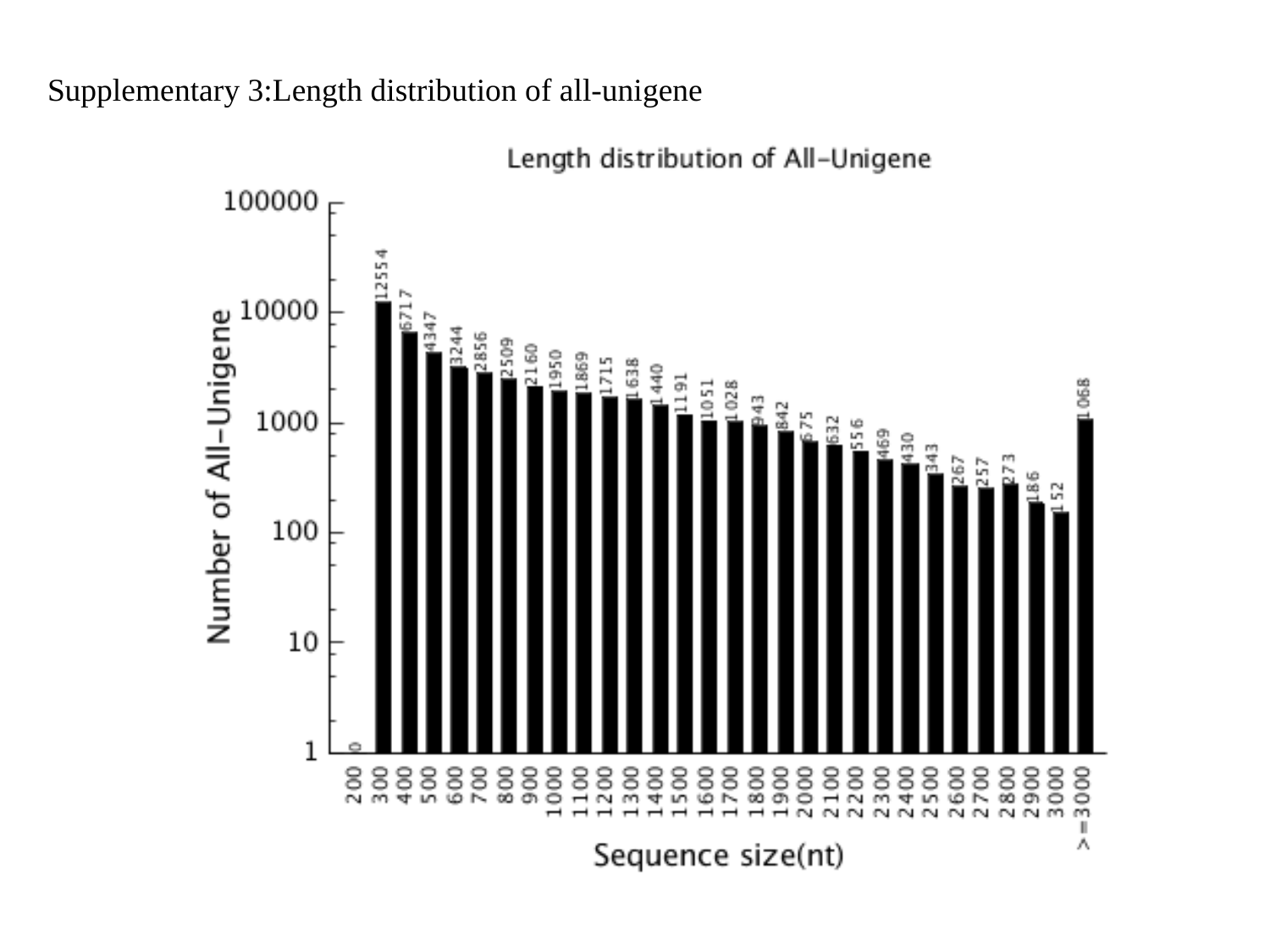

Supplementary 3:Length distribution of all-unigene

Supplement: Supplementary file 3 — 10.1186/s40709-016-0038-7 Length distribution of all-unigene extracted from S. linearistipularis. [file 40709_2016_38_MOESM3_ESM.pptx]

## Slide 1
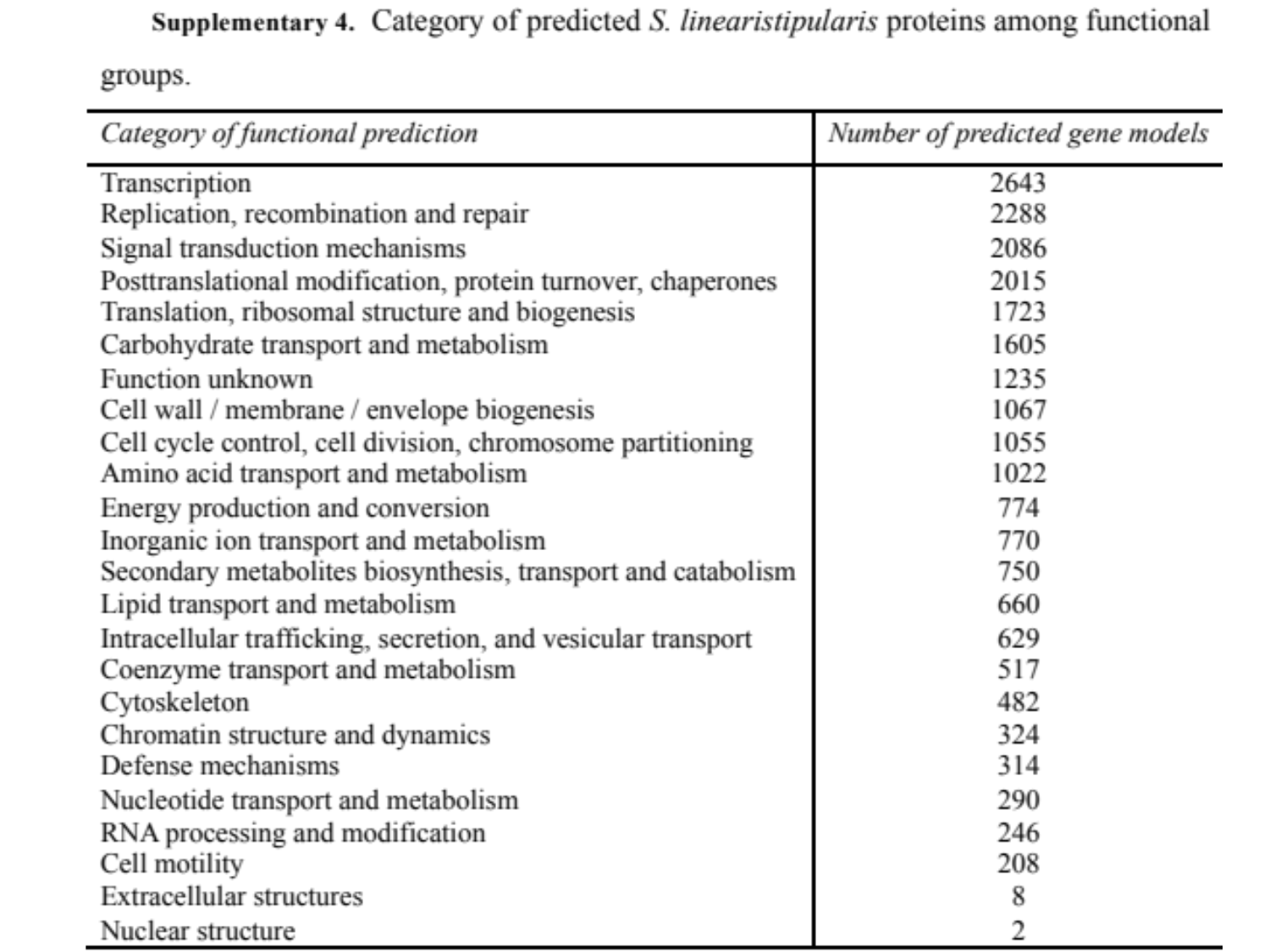

Supplement: Supplementary file 4 — 10.1186/s40709-016-0038-7 Category of predicted S. linearistipularis proteins among functional groups. [file 40709_2016_38_MOESM4_ESM.pptx]

## Slide 1
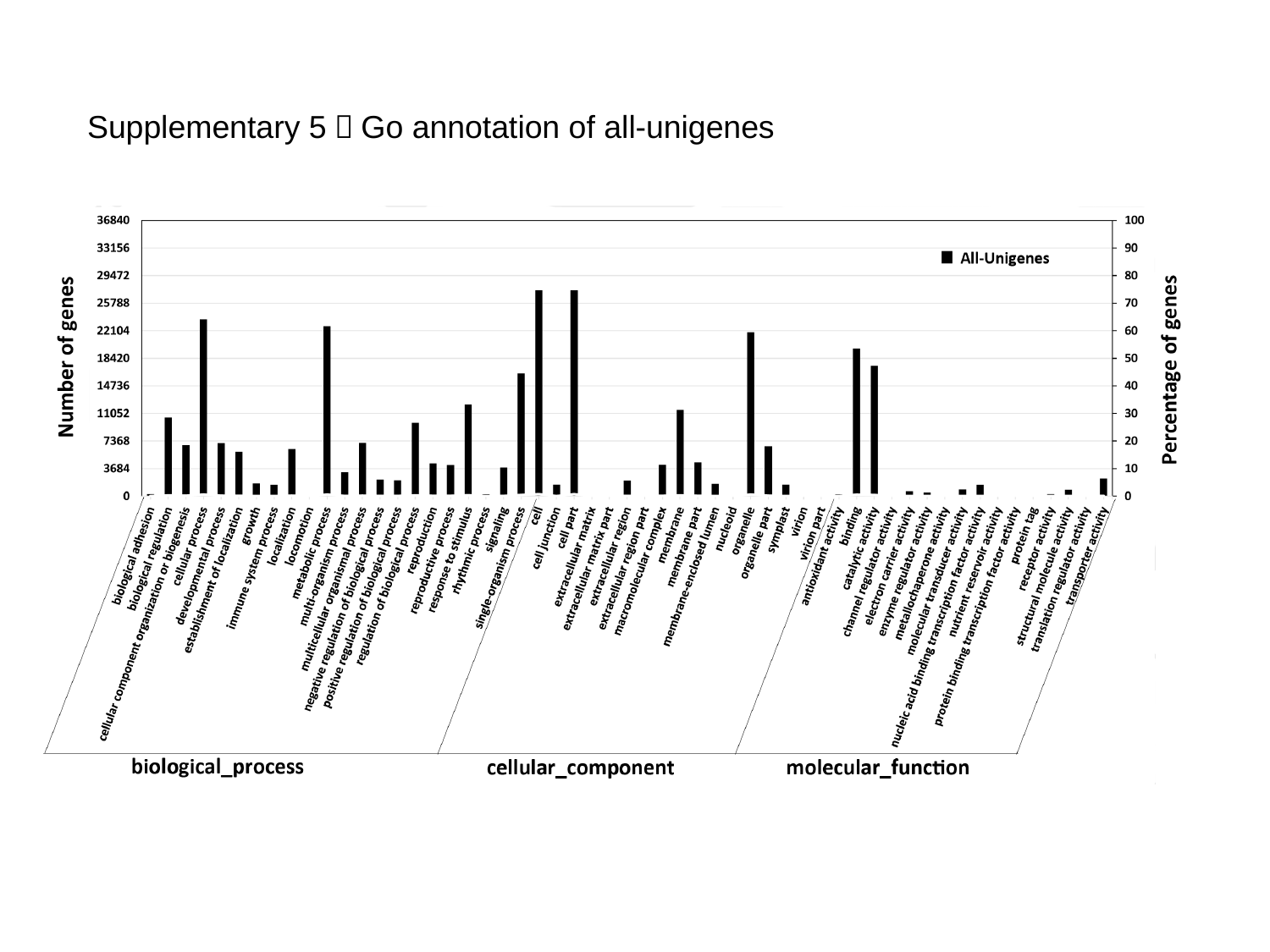

Supplementary 5：Go annotation of all-unigenes

Supplement: Supplementary file 5 — 10.1186/s40709-016-0038-7 Go annotation of S. linearistipularis all-unigenes. [file 40709_2016_38_MOESM5_ESM.pptx]
