# Supplementary material for: NaCl stress-induced transcriptomics analysis of Salix linearistipularis (syn. Salix mongolica)
Source: J Biol Res (Thessalon). 2016 Feb 29;23:1. doi: 10.1186/s40709-016-0038-7 (PMC4772304; doi:10.1186/s40709-016-0038-7)
Supplement: Supplementary file 6 — 10.1186/s40709-016-0038-7 Pathway annotation of S. linearistipularis all-unigenes. [file 40709_2016_38_MOESM6_ESM.ppt]

## Slide 1
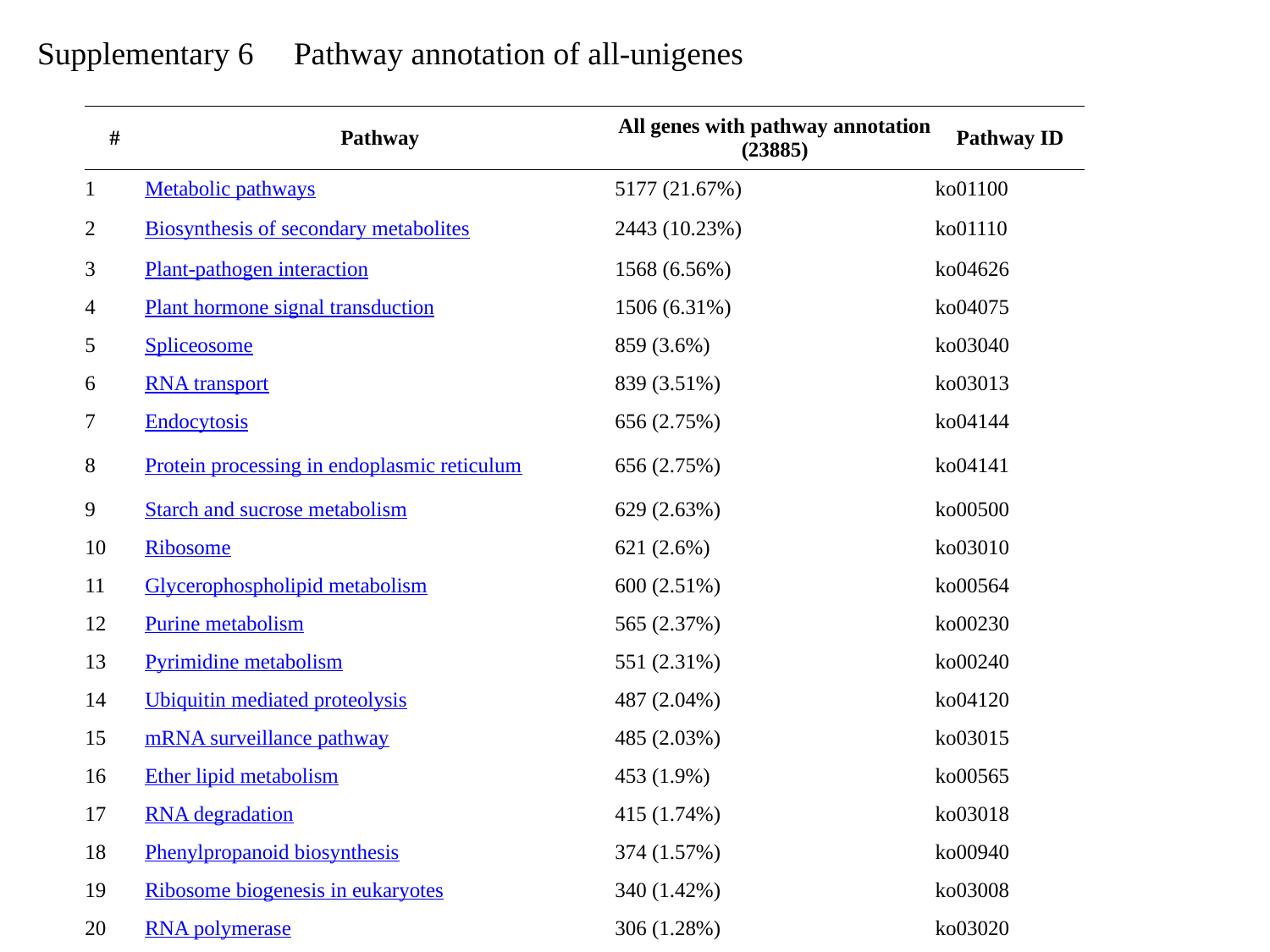

Supplementary 6：Pathway annotation of all-unigenes
| # | Pathway | All genes with pathway annotation (23885) | Pathway ID |
| --- | --- | --- | --- |
| 1 | Metabolic pathways | 5177 (21.67%) | ko01100 |
| 2 | Biosynthesis of secondary metabolites | 2443 (10.23%) | ko01110 |
| 3 | Plant-pathogen interaction | 1568 (6.56%) | ko04626 |
| 4 | Plant hormone signal transduction | 1506 (6.31%) | ko04075 |
| 5 | Spliceosome | 859 (3.6%) | ko03040 |
| 6 | RNA transport | 839 (3.51%) | ko03013 |
| 7 | Endocytosis | 656 (2.75%) | ko04144 |
| 8 | Protein processing in endoplasmic reticulum | 656 (2.75%) | ko04141 |
| 9 | Starch and sucrose metabolism | 629 (2.63%) | ko00500 |
| 10 | Ribosome | 621 (2.6%) | ko03010 |
| 11 | Glycerophospholipid metabolism | 600 (2.51%) | ko00564 |
| 12 | Purine metabolism | 565 (2.37%) | ko00230 |
| 13 | Pyrimidine metabolism | 551 (2.31%) | ko00240 |
| 14 | Ubiquitin mediated proteolysis | 487 (2.04%) | ko04120 |
| 15 | mRNA surveillance pathway | 485 (2.03%) | ko03015 |
| 16 | Ether lipid metabolism | 453 (1.9%) | ko00565 |
| 17 | RNA degradation | 415 (1.74%) | ko03018 |
| 18 | Phenylpropanoid biosynthesis | 374 (1.57%) | ko00940 |
| 19 | Ribosome biogenesis in eukaryotes | 340 (1.42%) | ko03008 |
| 20 | RNA polymerase | 306 (1.28%) | ko03020 |
| 21 | ABC transporters | 305 (1.28%) | ko02010 |
| 22 | Amino sugar and nucleotide sugar metabolism | 297 (1.24%) | ko00520 |
| 23 | Oxidative phosphorylation | 273 (1.14%) | ko00190 |
| 24 | Circadian rhythm - plant | 272 (1.14%) | ko04712 |

## Slide 2
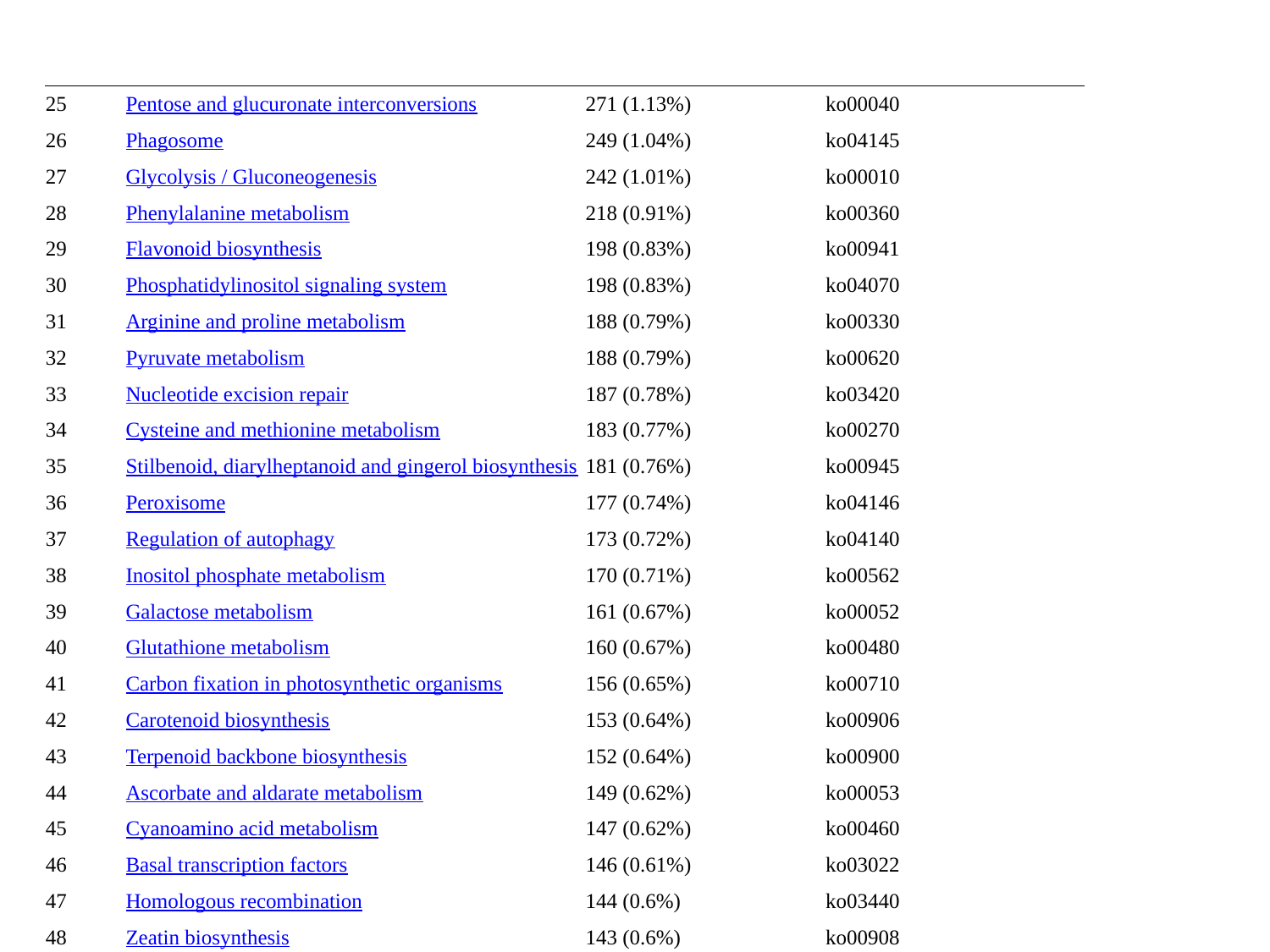

| 25 | Pentose and glucuronate interconversions | 271 (1.13%) | ko00040 |
| --- | --- | --- | --- |
| 26 | Phagosome | 249 (1.04%) | ko04145 |
| 27 | Glycolysis / Gluconeogenesis | 242 (1.01%) | ko00010 |
| 28 | Phenylalanine metabolism | 218 (0.91%) | ko00360 |
| 29 | Flavonoid biosynthesis | 198 (0.83%) | ko00941 |
| 30 | Phosphatidylinositol signaling system | 198 (0.83%) | ko04070 |
| 31 | Arginine and proline metabolism | 188 (0.79%) | ko00330 |
| 32 | Pyruvate metabolism | 188 (0.79%) | ko00620 |
| 33 | Nucleotide excision repair | 187 (0.78%) | ko03420 |
| 34 | Cysteine and methionine metabolism | 183 (0.77%) | ko00270 |
| 35 | Stilbenoid, diarylheptanoid and gingerol biosynthesis | 181 (0.76%) | ko00945 |
| 36 | Peroxisome | 177 (0.74%) | ko04146 |
| 37 | Regulation of autophagy | 173 (0.72%) | ko04140 |
| 38 | Inositol phosphate metabolism | 170 (0.71%) | ko00562 |
| 39 | Galactose metabolism | 161 (0.67%) | ko00052 |
| 40 | Glutathione metabolism | 160 (0.67%) | ko00480 |
| 41 | Carbon fixation in photosynthetic organisms | 156 (0.65%) | ko00710 |
| 42 | Carotenoid biosynthesis | 153 (0.64%) | ko00906 |
| 43 | Terpenoid backbone biosynthesis | 152 (0.64%) | ko00900 |
| 44 | Ascorbate and aldarate metabolism | 149 (0.62%) | ko00053 |
| 45 | Cyanoamino acid metabolism | 147 (0.62%) | ko00460 |
| 46 | Basal transcription factors | 146 (0.61%) | ko03022 |
| 47 | Homologous recombination | 144 (0.6%) | ko03440 |
| 48 | Zeatin biosynthesis | 143 (0.6%) | ko00908 |
| 49 | Glycerolipid metabolism | 141 (0.59%) | ko00561 |
| 50 | Alanine, aspartate and glutamate metabolism | 138 (0.58%) | ko00250 |

## Slide 3
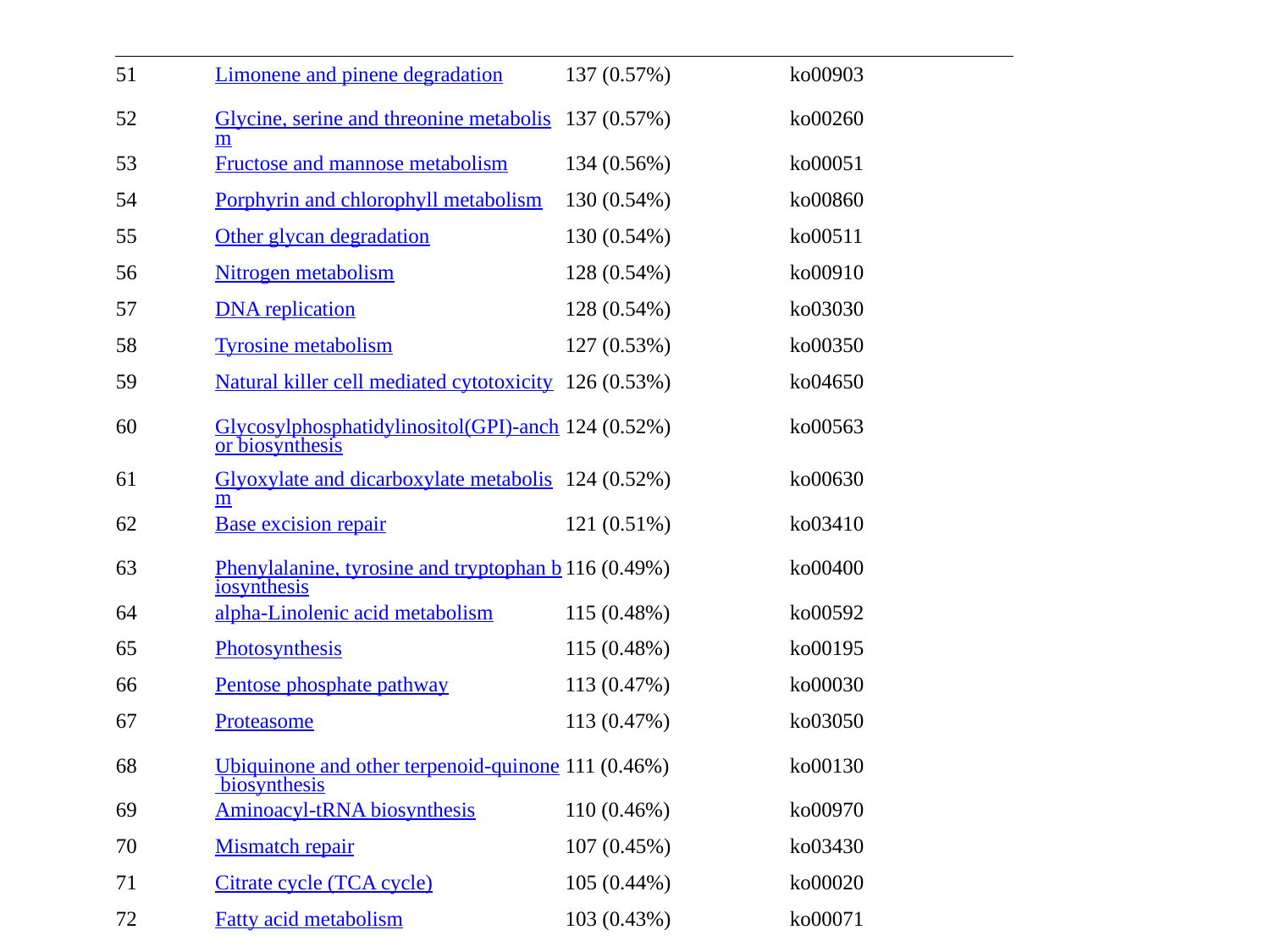

| 51 | Limonene and pinene degradation | 137 (0.57%) | ko00903 |
| --- | --- | --- | --- |
| 52 | Glycine, serine and threonine metabolism | 137 (0.57%) | ko00260 |
| 53 | Fructose and mannose metabolism | 134 (0.56%) | ko00051 |
| 54 | Porphyrin and chlorophyll metabolism | 130 (0.54%) | ko00860 |
| 55 | Other glycan degradation | 130 (0.54%) | ko00511 |
| 56 | Nitrogen metabolism | 128 (0.54%) | ko00910 |
| 57 | DNA replication | 128 (0.54%) | ko03030 |
| 58 | Tyrosine metabolism | 127 (0.53%) | ko00350 |
| 59 | Natural killer cell mediated cytotoxicity | 126 (0.53%) | ko04650 |
| 60 | Glycosylphosphatidylinositol(GPI)-anchor biosynthesis | 124 (0.52%) | ko00563 |
| 61 | Glyoxylate and dicarboxylate metabolism | 124 (0.52%) | ko00630 |
| 62 | Base excision repair | 121 (0.51%) | ko03410 |
| 63 | Phenylalanine, tyrosine and tryptophan biosynthesis | 116 (0.49%) | ko00400 |
| 64 | alpha-Linolenic acid metabolism | 115 (0.48%) | ko00592 |
| 65 | Photosynthesis | 115 (0.48%) | ko00195 |
| 66 | Pentose phosphate pathway | 113 (0.47%) | ko00030 |
| 67 | Proteasome | 113 (0.47%) | ko03050 |
| 68 | Ubiquinone and other terpenoid-quinone biosynthesis | 111 (0.46%) | ko00130 |
| 69 | Aminoacyl-tRNA biosynthesis | 110 (0.46%) | ko00970 |
| 70 | Mismatch repair | 107 (0.45%) | ko03430 |
| 71 | Citrate cycle (TCA cycle) | 105 (0.44%) | ko00020 |
| 72 | Fatty acid metabolism | 103 (0.43%) | ko00071 |
| 73 | SNARE interactions in vesicular transport | 101 (0.42%) | ko04130 |
| 74 | Propanoate metabolism | 100 (0.42%) | ko00640 |
| 75 | Protein export | 99 (0.41%) | ko03060 |

## Slide 4
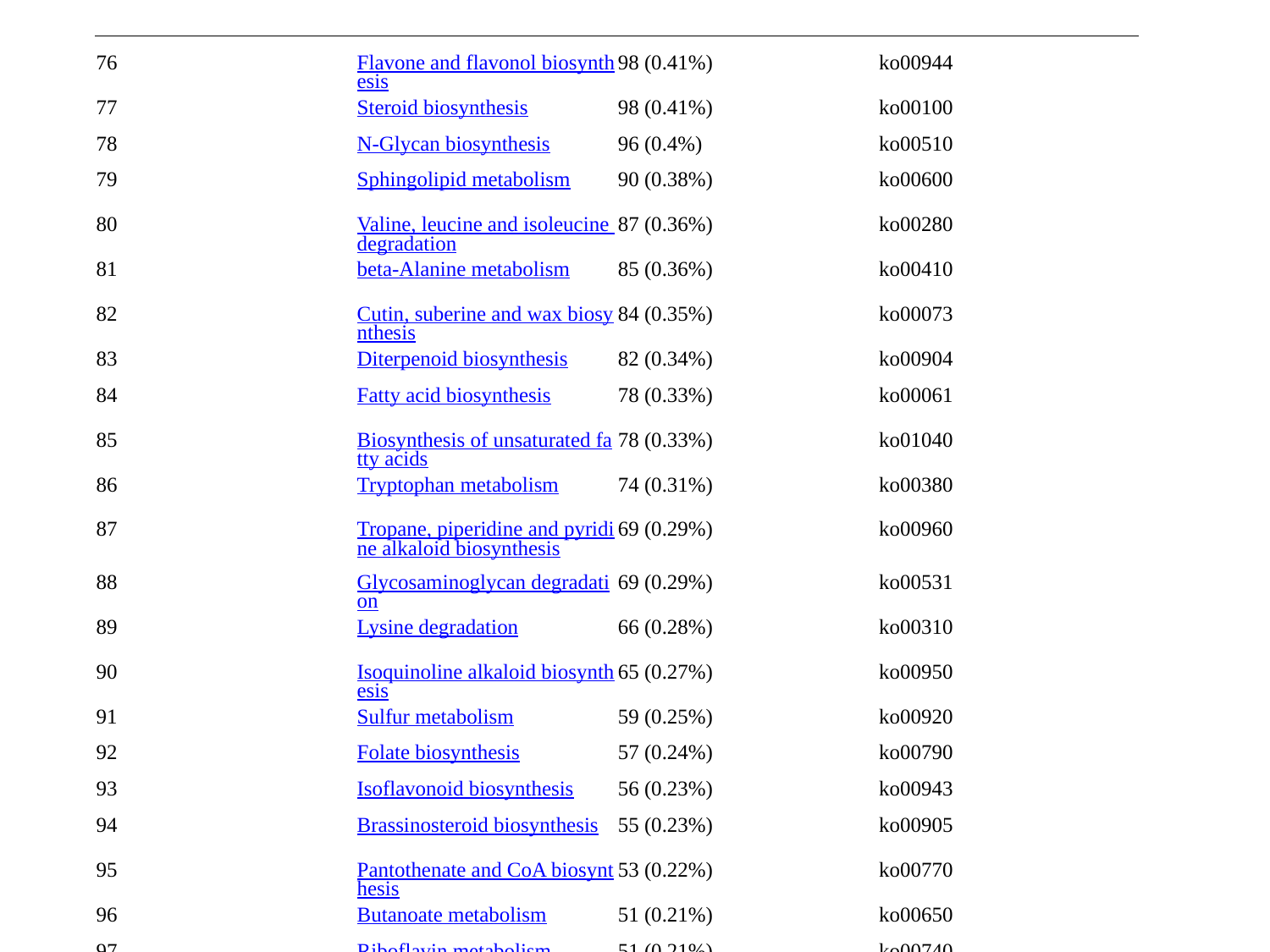

| 76 | Flavone and flavonol biosynthesis | 98 (0.41%) | ko00944 |
| --- | --- | --- | --- |
| 77 | Steroid biosynthesis | 98 (0.41%) | ko00100 |
| 78 | N-Glycan biosynthesis | 96 (0.4%) | ko00510 |
| 79 | Sphingolipid metabolism | 90 (0.38%) | ko00600 |
| 80 | Valine, leucine and isoleucine degradation | 87 (0.36%) | ko00280 |
| 81 | beta-Alanine metabolism | 85 (0.36%) | ko00410 |
| 82 | Cutin, suberine and wax biosynthesis | 84 (0.35%) | ko00073 |
| 83 | Diterpenoid biosynthesis | 82 (0.34%) | ko00904 |
| 84 | Fatty acid biosynthesis | 78 (0.33%) | ko00061 |
| 85 | Biosynthesis of unsaturated fatty acids | 78 (0.33%) | ko01040 |
| 86 | Tryptophan metabolism | 74 (0.31%) | ko00380 |
| 87 | Tropane, piperidine and pyridine alkaloid biosynthesis | 69 (0.29%) | ko00960 |
| 88 | Glycosaminoglycan degradation | 69 (0.29%) | ko00531 |
| 89 | Lysine degradation | 66 (0.28%) | ko00310 |
| 90 | Isoquinoline alkaloid biosynthesis | 65 (0.27%) | ko00950 |
| 91 | Sulfur metabolism | 59 (0.25%) | ko00920 |
| 92 | Folate biosynthesis | 57 (0.24%) | ko00790 |
| 93 | Isoflavonoid biosynthesis | 56 (0.23%) | ko00943 |
| 94 | Brassinosteroid biosynthesis | 55 (0.23%) | ko00905 |
| 95 | Pantothenate and CoA biosynthesis | 53 (0.22%) | ko00770 |
| 96 | Butanoate metabolism | 51 (0.21%) | ko00650 |
| 97 | Riboflavin metabolism | 51 (0.21%) | ko00740 |
| 98 | One carbon pool by folate | 48 (0.2%) | ko00670 |
| 99 | Fatty acid elongation | 46 (0.19%) | ko00062 |
| 100 | Selenocompound metabolism | 45 (0.19%) | ko00450 |

## Slide 5
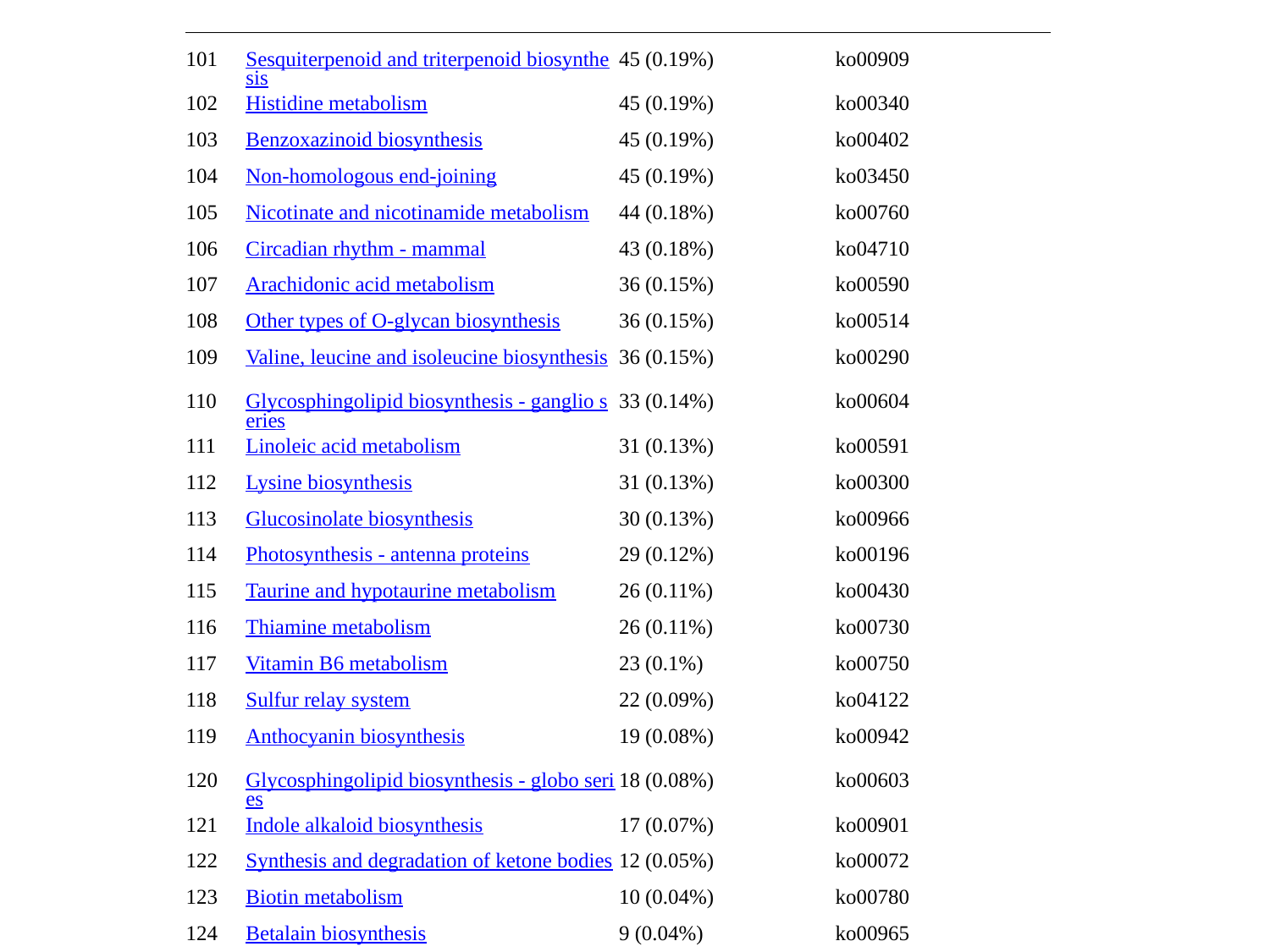

| 101 | Sesquiterpenoid and triterpenoid biosynthesis | 45 (0.19%) | ko00909 |
| --- | --- | --- | --- |
| 102 | Histidine metabolism | 45 (0.19%) | ko00340 |
| 103 | Benzoxazinoid biosynthesis | 45 (0.19%) | ko00402 |
| 104 | Non-homologous end-joining | 45 (0.19%) | ko03450 |
| 105 | Nicotinate and nicotinamide metabolism | 44 (0.18%) | ko00760 |
| 106 | Circadian rhythm - mammal | 43 (0.18%) | ko04710 |
| 107 | Arachidonic acid metabolism | 36 (0.15%) | ko00590 |
| 108 | Other types of O-glycan biosynthesis | 36 (0.15%) | ko00514 |
| 109 | Valine, leucine and isoleucine biosynthesis | 36 (0.15%) | ko00290 |
| 110 | Glycosphingolipid biosynthesis - ganglio series | 33 (0.14%) | ko00604 |
| 111 | Linoleic acid metabolism | 31 (0.13%) | ko00591 |
| 112 | Lysine biosynthesis | 31 (0.13%) | ko00300 |
| 113 | Glucosinolate biosynthesis | 30 (0.13%) | ko00966 |
| 114 | Photosynthesis - antenna proteins | 29 (0.12%) | ko00196 |
| 115 | Taurine and hypotaurine metabolism | 26 (0.11%) | ko00430 |
| 116 | Thiamine metabolism | 26 (0.11%) | ko00730 |
| 117 | Vitamin B6 metabolism | 23 (0.1%) | ko00750 |
| 118 | Sulfur relay system | 22 (0.09%) | ko04122 |
| 119 | Anthocyanin biosynthesis | 19 (0.08%) | ko00942 |
| 120 | Glycosphingolipid biosynthesis - globo series | 18 (0.08%) | ko00603 |
| 121 | Indole alkaloid biosynthesis | 17 (0.07%) | ko00901 |
| 122 | Synthesis and degradation of ketone bodies | 12 (0.05%) | ko00072 |
| 123 | Biotin metabolism | 10 (0.04%) | ko00780 |
| 124 | Betalain biosynthesis | 9 (0.04%) | ko00965 |
| 125 | Lipoic acid metabolism | 8 (0.03%) | ko00785 |
| 126 | Monoterpenoid biosynthesis | 8 (0.03%) | ko00902 |
| 127 | C5-Branched dibasic acid metabolism | 7 (0.03%) | ko00660 |
| 128 | Caffeine metabolism | 2 (0.01%) | ko00232 |
