# Supplementary material for: NaCl stress-induced transcriptomics analysis of Salix linearistipularis (syn. Salix mongolica)
Source: J Biol Res (Thessalon). 2016 Feb 29;23:1. doi: 10.1186/s40709-016-0038-7 (PMC4772304; doi:10.1186/s40709-016-0038-7)
Supplement: Supplementary file 7 — 10.1186/s40709-016-0038-7 qRT-PCR Primer design. [file 40709_2016_38_MOESM7_ESM.pptx]

## Slide 1
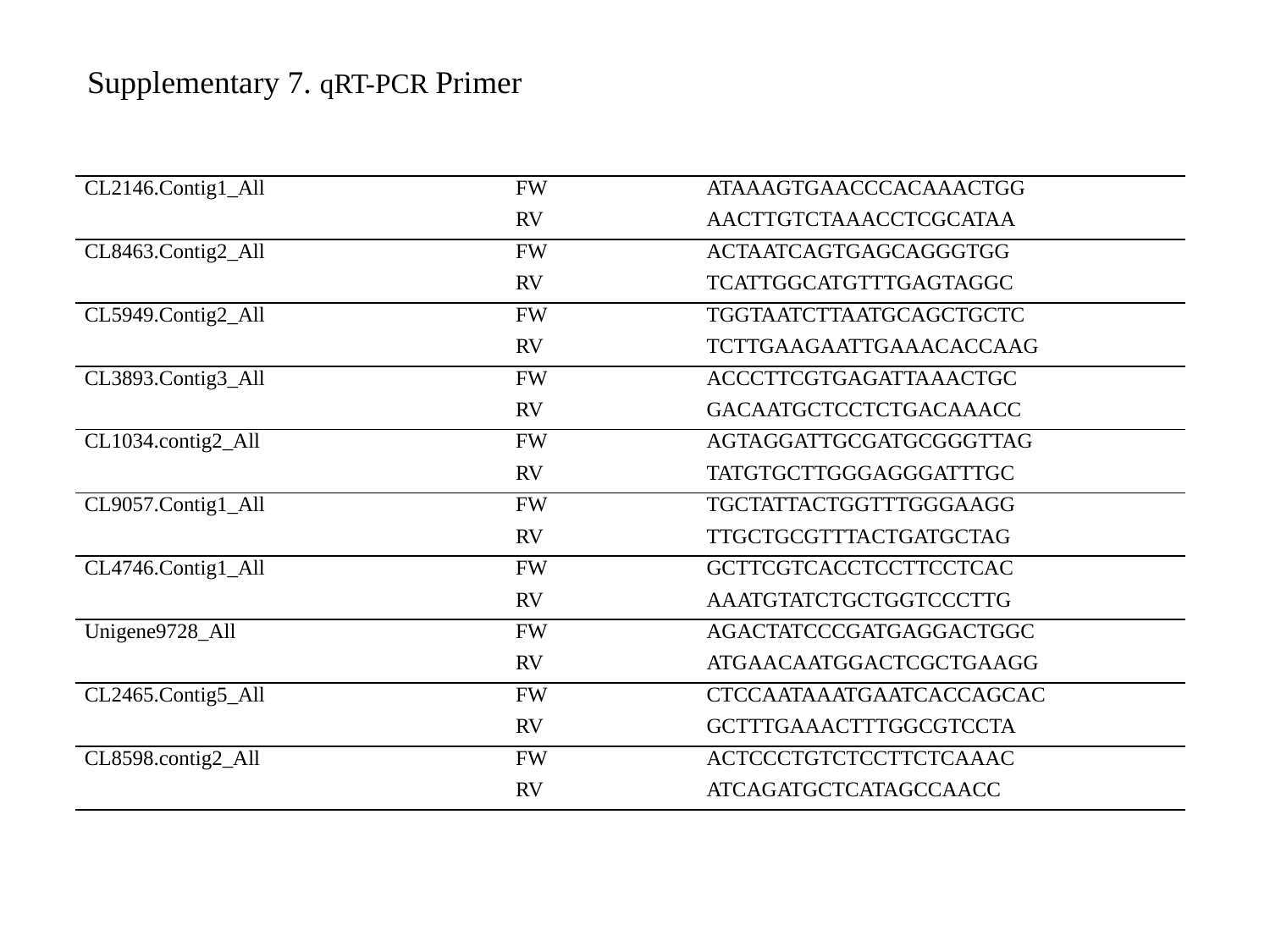

Supplementary 7. qRT-PCR Primer
| CL2146.Contig1\_All | FW | ATAAAGTGAACCCACAAACTGG |
| --- | --- | --- |
| | RV | AACTTGTCTAAACCTCGCATAA |
| CL8463.Contig2\_All | FW | ACTAATCAGTGAGCAGGGTGG |
| | RV | TCATTGGCATGTTTGAGTAGGC |
| CL5949.Contig2\_All | FW | TGGTAATCTTAATGCAGCTGCTC |
| | RV | TCTTGAAGAATTGAAACACCAAG |
| CL3893.Contig3\_All | FW | ACCCTTCGTGAGATTAAACTGC |
| | RV | GACAATGCTCCTCTGACAAACC |
| CL1034.contig2\_All | FW | AGTAGGATTGCGATGCGGGTTAG |
| | RV | TATGTGCTTGGGAGGGATTTGC |
| CL9057.Contig1\_All | FW | TGCTATTACTGGTTTGGGAAGG |
| | RV | TTGCTGCGTTTACTGATGCTAG |
| CL4746.Contig1\_All | FW | GCTTCGTCACCTCCTTCCTCAC |
| | RV | AAATGTATCTGCTGGTCCCTTG |
| Unigene9728\_All | FW | AGACTATCCCGATGAGGACTGGC |
| | RV | ATGAACAATGGACTCGCTGAAGG |
| CL2465.Contig5\_All | FW | CTCCAATAAATGAATCACCAGCAC |
| | RV | GCTTTGAAACTTTGGCGTCCTA |
| CL8598.contig2\_All | FW | ACTCCCTGTCTCCTTCTCAAAC |
| | RV | ATCAGATGCTCATAGCCAACC |
